# Twenty-eight new species of *Trigonopterus* Fauvel (Coleoptera, Curculionidae) from Central Sulawesi

**DOI:** 10.3897/zookeys.1065.71680

**Published:** 2021-10-22

**Authors:** Raden Pramesa Narakusumo, Alexander Riedel

**Affiliations:** 1 Museum of Natural History Karlsruhe, Erbprinzenstr. 13, D-76133 Karlsruhe, Germany Museum of Natural History Karlsruhe Karlsruhe Germany; 2 Museum Zoologicum Bogoriense, Research Center for Biology, Indonesian Institute of Sciences (LIPI), Jl. Raya Jakarta – Bogor, KM46, 16911, Indonesia Museum Zoologicum Bogoriense Raya Jakarta – Bogor Indonesia

**Keywords:** Celebes, conservation, *cox1*, Cryptorhynchinae, DNA barcoding, endemism, hyperdiverse, integrative taxonomy, morphology, Southeast Asia, turbo-taxonomy, Wallacea

## Abstract

Here we present 28 new species of *Trigonopterus* from Central Sulawesi, mostly from Mt Dako and Mt Pompangeo: *Trigonopterusacutus***sp. nov.**, *T.ancora***sp. nov.**, *T.arcanus***sp. nov.**, *T.corona***sp. nov.**, *T.dakoensis***sp. nov.**, *T.daun***sp. nov.**, *T.ewok***sp. nov.**, *T.gundala***sp. nov.**, *T.hoppla***sp. nov.**, *T.kakimerah***sp. nov.**, *T.katopasensis***sp. nov.**, *T.matakensis***sp. nov.**, *T.moduai***sp. nov.**, *T.mons***sp. nov.**, *T.paramoduai***sp. nov.***T.pomberimbensis***sp. nov.**, *T.pompangeensis***sp. nov.**, *T.puspoi***sp. nov.**, *T.rosichoni***sp. nov.**, *T.rubidus***sp. nov.**, *T.sarinoi***sp. nov.**, *T.sutrisnoi***sp. nov.**, *T.tanah***sp. nov.**, *T.tejokusumoi***sp. nov.**, *T.toboliensis***sp. nov.**, *T.tolitoliensis***sp. nov.**, *T.tounaensis***sp. nov.**, *T.unyil***sp. nov.** This fills important areas of distribution and brings the number of *Trigonopterus* species recorded from Sulawesi to 132.

## Introduction

*Trigonopterus* is a hyperdiverse genus of flightless hidden-snout weevils (Cryptorhynchinae) ranging over the Indo-Australian-Melanesian archipelago. It originated in Northern Australia and rapidly diversified in New Guinea (Toussaint et al. 2017) before colonizing Sulawesi and dispersing further west to Sundaland (Tänzler et al. 2016; Letsch et al. 2020). Thus, Sulawesi acted as a hub for the dispersal to Borneo, Java and the Lesser Sunda Islands. Currently, there are 451 described species (Riedel et al. 2013b, 2014; Riedel and Tänzler 2016; Narakusumo et al. 2019; Riedel and Narakusumo 2019), but discovery of new species is still far from approaching saturation, especially if new localities are being sampled. In the following, we report on 28 new species from Central Sulawesi Province, mostly based on two field trips to Mt Dako and Mt Pompangeo, plus four species from Mt Torompupu, Mt Katopasa, and Palu from the collection of “Museum Zoologicum Bogoriense”. Mt Dako with a maximum elevation of 2304 m was sampled between 700 and 2200 m. Mt Pompangeo with a maximum elevation of 2590 m was sampled between 1800 and 2000 m. The total of *Trigonopterus* species in Sulawesi and the adjacent islands was recently brought from a single one to 104 species (Riedel and Narakusumo 2019), and with the present paper to 132 species. We refrain from providing a key based on morphological characters for the same reasons as outlined previously (Riedel and Narakusumo 2019: p. 96). Until the number of described species approaches saturation, a traditional key would be incomplete and potentially highly misleading. Old museum specimens can be hard to identify based on *cox1* sequences using a PCR / Sanger sequencing workflow, but in many cases, fragments of degraded DNA will be sufficient to allow sequencing by NGS technologies (Staats et al. 2013). Thus, even dry specimens older than 100 years can be safely identified if deemed necessary.

Sulawesi is geologically complex (Hall 2009; Stelbrink et al. 2012) and its biogeography is currently the subject of a detailed study by us utilizing, among other taxa, the genus *Trigonopterus*. The purpose of this paper is to provide names to these species, especially as some of them had been part of an earlier study on mitogenomes (Narakusumo et. al. 2020). While many additional new *Trigonopterus* species can be expected from Sulawesi, the species described herein fill an important gap in the distributional record (Fig. 29). Central Sulawesi is where the formerly separate geological terranes fused together (Hall 2009; Nugraha and Hall 2018), and its fauna may be one of the richest areas on this island.

## Materials and methods

This study is based on 866 specimens from Central Sulawesi Province. Holotypes were selected from 197 specimens for which the *cox1* gene had been sequenced. DNA was extracted nondestructively as described by Riedel et al. (2010). Genitalia of most specimens did not require extra maceration. They could be directly stained with an 0.01% alcoholic Chlorazol Black solution and stored in glycerol in microvials attached to the pin of the specimens. Genitalia of specimens with tissue not sufficiently digested after DNA extraction were macerated in a 10% KOH solution and rinsed in 5% acetic acid before staining. Illustrations of habitus and genitalia were prepared from holotypes. Finally, type series were supplemented with specimens stored in ethanol and older material from the dry collection. Long series of the sibling species *T.matakensis* sp. nov. and *T.pompangeensis* sp. nov. could not be assigned based on external characters and had to remain unidentified. Type depositories are cited using the following codens:

**MZB**LIPI Research Center of Biology, Division of Zoology, Museum Zoologicum Bogoriense, Widyasatwaloka, Cibinong, Indonesia.

**SMNK**Staatliches Museum für Naturkunde, Karlsruhe, Germany.

The methods applied for DNA sequencing and sequence analysis are the same as described by Riedel et al. (2010) and Tänzler et al. (2012), except for samples MZB0217-MZB0240 being sequenced only in reverse direction using the primer HCO. Morphological descriptions are limited to major diagnostic characters as outlined by Riedel et al. (2013a, b). Negative character states (i.e., the absence of a character) are only mentioned explicitly where it appears appropriate. In groups comprising hundreds of species enumerating the absence of rare character states leads to inflated descriptions that distract the reader from the important information, i.e., the diagnostic characters present in a given species.

The closest relatives of Central Sulawesi species were identified by creating an alignment of 773 *cox1* sequences representing ca. 185 species and generating a maximum likelihood reconstruction using the program IQTREE (Nguyen et al. 2015). The uncorrected p-distance was calculated using dist.dna function with parameter model=”raw” and pairwise.deletion=“TRUE”, in ape 5.0 package (Paradis and Schliep 2019) run on R 3.6.3 (R Core Team 2020). Morphological terminology follows Beutel and Leschen (2005) and Leschen et al. (2009), i.e., the terms “mesoventrite” / “metaventrite” are used instead of “mesosternite” / “metasternite” and “mesanepisternum” / “metanepisternum” instead of “mesepisternum” / “metepisternum”; “penis” is used instead of “aedeagus” as the tegmen is usually without useful characters in *Trigonopterus* and therefore omitted from species descriptions. Specimens were examined with a Leica MZ16 dissecting microscope and a fluorescent desk lamp for illumination. Measurements were taken with the help of an ocular grid. The length of the body was measured in dorsal aspect from the elytral apex to the front of the pronotum. Legs were described in an idealized laterally extended position; there is a dorsal / ventral and an anterior / posterior surface. Habitus illustrations were compiled using a DFC495 camera with L.A.S. 4.8.0 software adapted to a Z6 APO (all from Leica Microsystems, Heerbrugg, Switzerland). Photographic illustrations of genitalia were made using a DFC450 camera with L.A.S. 4.8.0 software adapted to an Axio Imager M2 microscope (Carl Zeiss Microscopy), with 5×, respectively 10× A-Plan lenses; resulting image stacks were compiled using the Helicon Focus 6.7.1 Pro software (Helicon Soft Ltd). For photography genitalia were temporarily embedded in glycerol gelatin as described by Riedel (2005), with their longitudinal axis somewhat lifted caudally, to adequately illustrate structures of the curved down apex. All photographs were enhanced using the programs Adobe Photoshop CS2 and CS6. However, care was taken not to obscure or alter any features of the specimens illustrated. Sequence data were submitted to GenBank of NCBI (National Center for Biotechnology Information) and the accession numbers are provided under each species, e.g., as “(GenBank OK481808)”.

## Taxonomy

### Trigonopterus

Taxon classificationAnimaliaColeopteraCurculionidae

Fauvel, 1862

4D24708B-C6FE-5EA1-9016-4A066A7C4E9D

Trigonopterus Fauvel, 1862 Type species: Trigonopterusinsignis Fauvel, 1862, by monotypy.

#### Diagnosis.

Fully apterous genus of Cryptorhynchinae. Length 1.5–6.0 mm. Rostrum in repose not reaching center of mesocoxa. Scutellar shield completely absent externally. Mesothoracic receptacle deep, posteriorly closed. Metanepisternum completely absent externally. Elytra with nine striae (sometimes superficially effaced). Tarsal claws minute. Usually body largely unclothed, without dense vestiture. For additional information, see http://species-id.net/wiki/Trigonopterus.

##### Descriptions of the species

### 1. Trigonopterus acutus sp. nov.

Taxon classificationAnimaliaColeopteraCurculionidae

1ED69D4F-7EB3-5915-A2C3-8977D9E32A42

http://zoobank.org/BEA6B28C-CB9B-4C5F-934A-A10BB3611B07

#### Diagnostic description.

**Figure 1.**
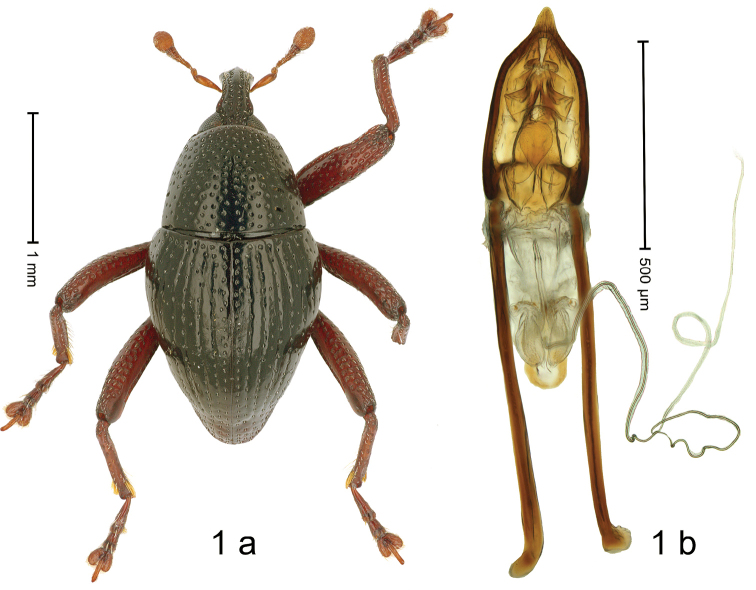
*Trigonopterusacutus* sp. nov., holotype **a** habitus **b** penis.

***Holotype*. Male** (Fig. 1a). Length 2.50 mm. Color of antennae and legs ferruginous; remainder black. Body subovate; in dorsal aspect and in profile with constriction between pronotum and elytron. Rostrum dorsally with median costa, and pair submedian ridge; intervening furrows with rows of coarse punctures and small suberect scales; epistome indistinct, subglabrous with suberect setae. Pronotum with disk densely punctate; interspaces between punctures subglabrous; laterally in basal half impunctate. Elytra with striae marked by well-impressed lines and rows of punctures; intervals subglabrous, with sparse punctures. Meso- and metafemur with anteroventral ridge crenate-denticulate. Metafemur subapically with stridulatory patch. Metatibia basally subglabrous, in apical half with few long setae. Abdominal ventrites 1–2 concave, anteriorly microreticulate, partly with coarse punctures, center subglabrous; ventrite 5 flat, densely punctate, microreticulate. Penis (Fig. 1b) with sides of body converging, apex acuminate, without setae; with several endophallic sclerites, especially around the ostium; with pair of triangular sclerites reinforcing basal orifice laterally; apodemes 2.0× as long as body of penis; transfer apparatus dentiform, curved upwards; ductus ejaculatorius with indistinct bulbus. ***Intraspecific variation***. Length 2.38–2.65 mm. Female rostrum slender, dorsally subglabrous, with rows of punctures.

#### Material examined.

***Holotype*** (MZB, Cole.173.053): MZB0160 (GenBank OK481878), Indonesia, C-Sulawesi, Toli-Toli, Gn. Dako, 01°02.977'N, 120°55.010'E to 01°03.210'N, 120°55.297'E, 1700–1800 m, 08–10-VII-2018, beaten. ***Paratypes*** (MZB, SMNK): Indonesia, C-Sulawesi, Toli-Toli, Gn. Dako: 5 exx, MZB0101 (GenBank OK481916), MZB0055 (GenBank OK481960), MZB0197 (GenBank OK481853), MZB0198 (GenBank OK481852), MZB0199 (GenBank OK481851), 01°02.977'N, 120°55.001'E to 01°03.210'N, 120°55.297'E, 1700–1800 m, 08–10-VII-2018, beaten; 4 exx, MZB0191 (GenBank OK481859), 01°03.782'N, 120°53.934'E to 01°02.977'N, 120°55.001'E, 1250–1750 m, 11-VII-2018, beaten.

#### Distribution.

C-Sulawesi Prov. (Mt Dako). Elevation ca. 1700–1800 m.

#### Biology.

On foliage in montane forest.

#### Etymology.

The species name is the Latin adjective *acutus -a -um* (pointed, acute) and refers both to the elytral shape and the apex of the penis.

#### Notes.

*Trigonopterusacutus* sp. nov. was coded as “*Trigonopterus* sp. 1207”. This species belongs to the *T.tatorensis*-group. It is closely related to *T.daun* sp. nov., from which it can be distinguished by the pointed apex of the penis.

### 2. Trigonopterus ancora sp. nov.

Taxon classificationAnimaliaColeopteraCurculionidae

D8C3F5EC-745D-5E53-AAAF-7632C643F4CF

http://zoobank.org/74813024-DE34-4E66-B8FD-8DD6C8EC33C3

#### Diagnostic description.

**Figure 2.**
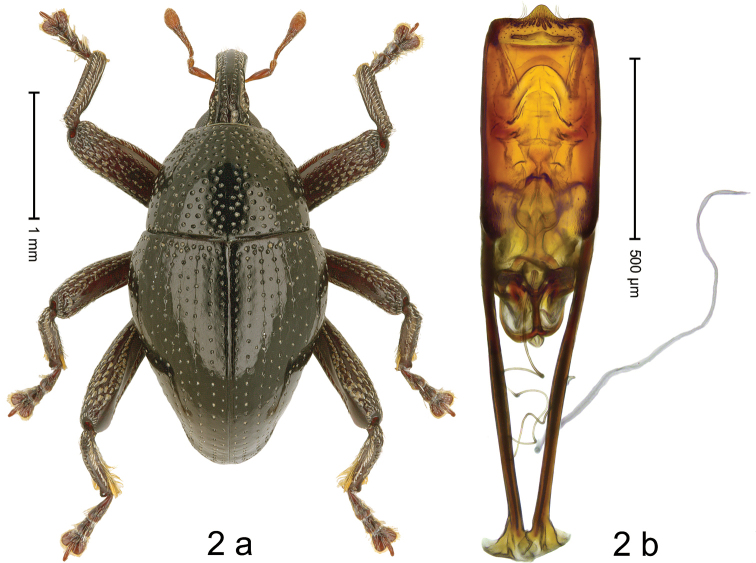
*Trigonopterusancora* sp. nov., holotype **a** habitus **b** penis.

***Holotype*,** male (Fig. 2a). Length 2.97 mm. Color of antennae ferruginous; legs dark ferruginous; remainder black. Body subovate; in dorsal aspect with weak constriction between pronotum and elytron; in profile dorsally convex. Rostrum dorsally with broad median costa and pair of submedian ridges; intervening furrows with sparse rows of recumbent setae; apical 1/3 subglabrous, with few punctures and with sparse setae. Pronotum with disk densely punctate with coarse punctures except along impunctate midline; interspaces between punctures subglabrous, subequal to or smaller than punctures´ diameter. Elytra with striae marked by punctures and fine hairlines; basal margin bordered by transverse row; intervals flat, with few interspersed punctures. Femora edentate; anteroventral ridges simple. Metafemur dorsally with sparse, recumbent, silvery scales; dorsoposterior edge crenate; subapically with stridulatory patch. Metatibia in apical 1/2 with fringe of yellow setae. Abdominal ventrites 1–2 concave, subglabrous; sublaterally sparsely punctate, with sparse setae; ventrite 5 with deeply concave impression of subquadrate outline; lateral ridges and subapically densely punctate, sparsely setose. Penis (Fig. 2b) with sides of body subparallel; apex subtruncate, with median angulate extension, with sparse setae; ventrolaterally at middle with pair of knobs; apodemes 1.8× as long as body of penis; transfer apparatus spiniform, directed basad in repose, attached to anchor-shaped supporting sclerite; ductus ejaculatorius without bulbus. ***Intraspecific variation***. Length 2.28–2.97 mm. Female rostrum dorsally flattened, smooth, with sublateral furrows and submedian rows of small punctures. Female abdominal ventrite 5 flat, subglabrous, laterally with sparse punctures.

#### Material examined.

***Holotype*** (MZB, Cole.173.054): MZB0053 (GenBank OK481962), Indonesia, C-Sulawesi, Toli-Toli, Gn. Dako, 01°03.412'N, 120°54.126'E to 01°03.241'N, 120°54.328'E, 1200–1300 m, 07-VII-2018, beaten. ***Paratypes*** (MZB, SMNK): Indonesia, C-Sulawesi, Toli-Toli, Gn. Dako: 1 ex, MZB0251 (GenBank OK481802), same data as holotype; 24 exx, MZB0054 (GenBank OK481961), MZB0258 (GenBank OK481795), 01°03.782N, 120°53.934'E to 01°03.574'N, 120°54.032'E, 970–1100 m, 05–06-VII-2018, beaten; 2 exx, 01°03.782'N, 120°53.934'E to 01°03.967N, 120°53.692'E, 970–1000 m, 05-VII-2018, beaten; 4 exx, MZB0247 (GenBank OK481806), 01°03.782N, 120°53.934'E, 970 m, 07-VII-2018, beaten; 5 exx, MZB0241 (GenBank OK481812), MZB0242 (GenBank OK481811), MZB0243 (GenBank OK481810), MZB0244 (GenBank OK481809), MZB0245 (GenBank OK481808), 01°03.782N, 120°53.934'E to 01°03.574N, 120°54.032'E, 970–1140 m, 06-VII-2018, beaten; 1 ex, MZB0057 (GenBank OK481958), 01°03.512'N, 120°54.054'E, 1100–1200 m, 13-VII-2018, sifted; 3 exx, MZB0250 (GenBank OK481803), MZB0256 (GenBank OK481797), MZB0257 (GenBank OK481796), 01°03.512'N, 120°54.054'E, 1100–1200 m, 06-VII-2018, beaten; 3 exx, same as holotype; 1 ex, MZB0248 (GenBank OK481805), 01°03.697'N, 120°53.991'E, 1030 m, 06-VII-2018, sifted; 1 ex, MZB0246 (GenBank OK481807), 01°03.574'N, 120°54.032'E to 01°03.181'N, 120°54.607'E, 1100–1400 m, 07-VII-2018, beaten; 4 exx, 01°03.241'N, 120°53.328'E to 01°03.181'N, 120°54.607'E, 1300–1400 m, 07-VII-2018, beaten; 1 ex, 01°03.014'N, 120°54.607'E to 01°02.977'N, 120°55.009'E, 1400–1750 m, 07-VII-2018, beaten.

#### Distribution.

C-Sulawesi Prov. (Mt Dako). Elevation 970–1400 m.

#### Biology.

On foliage and in leaf litter in montane forests.

#### Etymology.

This epithet is the Latin noun *ancora* (anchor) in apposition and refers to the sclerite in the male transfer apparatus.

#### Notes.

*Trigonopterusancora* sp. nov. was coded as “*Trigonopterus* sp. 1114” (Narakusumo et al. 2020). The species belongs to the *T.satyrus*-group and is closely related to *T.rosichoni* sp. nov. from which it differs by the shape of the transfer apparatus and 9.4–9.6% p-distance of its *cox1* sequence.

### 3. Trigonopterus arcanus sp. nov.

Taxon classificationAnimaliaColeopteraCurculionidae

76400314-7818-5E92-A7FB-E554771A341A

http://zoobank.org/88D3491B-F652-455A-80A4-CD8E683ACEDC

#### Diagnostic description.

**Figure 3.**
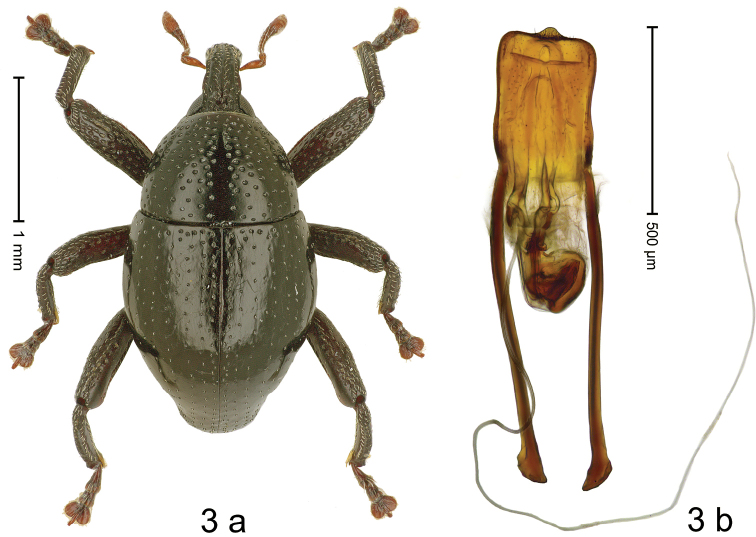
*Trigonopterusarcanus* sp. nov., holotype **a** habitus **b** Penis.

**Holotype**, male (Fig. 3a). Length 2.40 mm. Color of antennae ferruginous; remainder black. Body subovate; in dorsal aspect with weak constriction between pronotum and elytron; in profile dorsally convex. Rostrum dorsally with median ridge and pair of submedian ridges; intervening furrows with sparse rows of narrow, recumbent scales; epistome in apical 1/4 indistinct, subglabrous, with sparse setae. Pronotum with disk punctate, each puncture containing short seta; median line impunctate; interspaces subglabrous. Elytra with striae marked by rows of small punctures; intervals subglabrous; near base with few slightly larger punctures. Femora edentate; anteroventral ridge weakly crenate, ending in apical 1/3; anterior surface densely punctate, each puncture with narrow recumbent scale. Metafemur dorsally rounded, subapically with extensive stridulatory patch. Metatibia with dorsal edge weakly denticulate. Abdominal ventrites 1–2 weakly concave, subglabrous; ventrite 5 with dense coarse punctures, covered with suberect scales. Penis (Fig. 3b) with body subparallel, apex asymmetrical with subangulate extension, with few setae; apodemes 2.0× as long as body of penis; transfer apparatus complex, asymmetrical, with subrotund capsule; ductus ejaculatorius without bulbus. ***Intraspecific variation*.** Length 2.13–2.80 mm. Female rostrum dorsally polished, with submedian and sublateral rows of punctures. Female abdominal ventrite 1 weakly concave with sparse minute punctures. Female abdominal ventrite 5 flat, medially weakly convex, punctate, with suberect scales.

#### Material examined.

***Holotype*** (MZB, Cole.173.055): MZB0060 (GenBank OK481955), Indonesia, C-Sulawesi, Toli-Toli, Gn. Dako, 01°3.574'N, 120°54.0322'E to 01°03.180'N, 120°54.607'E 1100–1200 m, 06-VII-2018, beaten. ***Paratype*** (MZB, SMNK): Indonesia, C-Sulawesi, Toli-Toli, Gn. Dako: 2 exx, same data as holotype; 3 exx, MZB0195 (GenBank OK481855), 01°04.181'N, 120°53.565'E to 01°03.967'N, 120°53.692'E, 720–830 m, 01-VII-2018, beaten; 3 exx, 01°03.574'N, 120°54.032'E to 01°03.512'N, 120°54.054'E, 1100–1200 m, 06-VII-2018, beaten; 11 exx, 01°03.782'N, 120°53.934'E to 01°03.574'N, 120°54.032'E, 970–1100 m, 06-VII-2018, beaten; 3 exx, 01°03.782N, 120°53.934'E to 01°03.574N, 120°54.032'E, 970–1140 m, 06-VII-2018, beaten; 2 exx, 01°03.782'N, 120°53.934'E, 970 m, 04-VII-2018, beaten; 2 exx, MZB0281 (GenBank OK481775), 01°03.782'N, 120°53.934'E, 970 m, 07-VII-2018, beaten; 3 exx, 01°03.967'N, 120°53.692'E to 01°03.782'N, 120°53.934'E, 830–970 m, 01-VII-2018, beaten; 1 ex, MZB0192 (GenBank OK481858), 01°04.181'N, 120°53.565'E to 01°03.967'N, 120°53.692'E, 835–970 m, 01-VII-2018, beaten; 7 exx, 01°03.967'N, 120°53.692'E to 01°03.782'N, 120°53.934'E, 830–970 m, 05-VII-2018, beaten; 7 exx, MZB0282 (GenBank OK481774), 01°03.967'N, 120°53.692'E to 01°03.782'N, 120°53.934'E, 830–970 m, 03-VII-2018, beaten; 3 exx, 01°03.574'N, 120°54.032'E to 01°03.157'N, 120°54.195'E, 1100–1120 m, 06-VII-2018, beaten; 9 exx, MZB0058 (GenBank OK481957), MZB0090 (GenBank OK481927), MZB0196 (GenBank OK481854), 01°03.412'N, 120°54.126'E to 01°03.241'N, 120°54.328'E, 1200–1300 m, 07-VII-2018, beaten; 3 exx, MZB0061 (GenBank OK481954), MZB0091 (GenBank OK481926), MZB0280 (GenBank OK481776), 01°03.697N, 120°53.991'E, 1030 m, 06-VII-2018, beaten; 13 exx, 01°03.697'N, 120°53.991'E, 1030 m, 06-VII-2018, sifted; 1 ex, 01°03.157'N, 120°54.195'E, 1120 m, 06-VII-2018, sifted; 1 ex, MZB0283 (GenBank OK481773), 01°03.157'N, 120°54.195'E, 1120 m, 13-VII-2018, sifted; 9 exx, 01°03.574'N, 120°54.032'E to 01°03.181'N, 120°54.607'E, 1100–1400 m, 07-VII-2018, beaten; 5 exx, 01°03.014'N, 120°54.607'E to 01°02.977'N, 120°55.009'E, 1400–1750 m, 01-VII-2018, beaten.

#### Distribution.

C-Sulawesi Prov. (Mt Dako). Elevation 830–1400 m.

#### Biology.

On foliage in montane forest.

#### Etymology.

This epithet is based on the Latin adjective *arcanus -a*, -*um* (hidden) and it refers to its close similarity to its sibling species *T.ovatulus* Riedel and *T.pseudovatulus* Riedel.

#### Notes.

*Trigonopterusarcanus* sp. nov. was coded as “*Trigonopterus* sp. 1187”. The species belongs to the *T.ovatulus*-group. It is closely related to *T.pseudovatulus* Riedel from which it can be distinguished by the absence of a metatibial supra-uncal tooth. Furthermore, it differs by 12.7–13.8% p-distances of its *cox1* sequence.

### 4. Trigonopterus corona sp. nov.

Taxon classificationAnimaliaColeopteraCurculionidae

72D67E09-678B-568B-BAA6-E02C552E3D1E

http://zoobank.org/67F09DA3-B2E0-4018-B8E7-45FB830B6D59

#### Diagnostic description.

**Figure 4.**
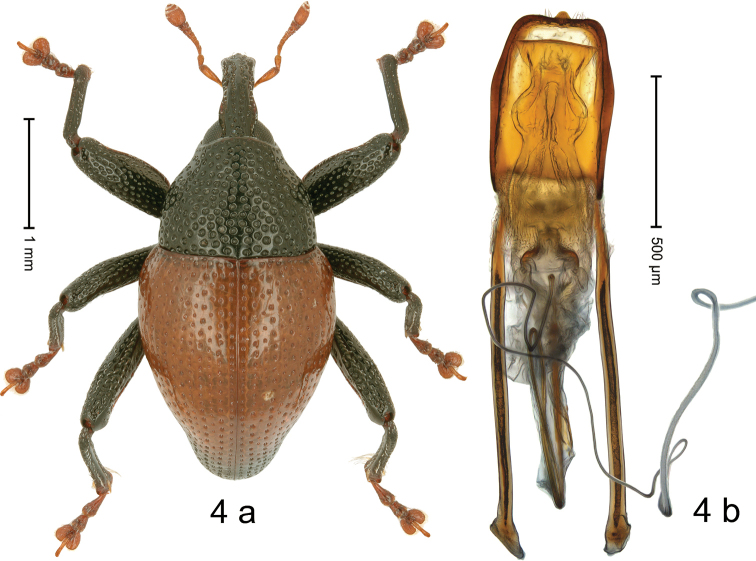
*Trigonopteruscorona* sp. nov., holotype **a** habitus **b** penis.

***Holotype*. Male** (Fig. 4a). Length 3.31 mm. Color of antennae, tarsi and elytra ferruginous; remainder black. Body subovate; in dorsal aspect with weak constriction between pronotum and elytron; in profile dorsally convex. Rostrum dorsally with median ridge and indistinct pair of submedian ridges, separated by rows of coarse punctures; epistome indistinct, subglabrous. Pronotum with disk densely punctate with coarse punctures; almost reticulate, interspaces subglabrous. Elytra ferruginous, apex darkened; striae marked by rows of larger punctures; intervals with rows of smaller punctures; stria 8 along humerus with six coarse punctures, externally bordered by indistinct ridge. Femora edentate, anteroventral ridge simple, crenulate; anterior surface densely coarsely punctate. Metafemur dorsally with sparse, subrecumbent, silvery scales; dorsoposterior edge simple; subapically with stridulatory patch. Metatibia in apical 1/3 ventrally with rounded, blade-like extension, with sparse, long setae. Abdominal ventrites 1–2 concave, sparsely punctate with coarse punctures, each puncture with one suberect silvery scale; ventrite 5 densely coarsely punctate, with suberect silvery scales, with shallow median impression. Penis (Fig. 4b) with sides basally subparallel, near middle angulate, weakly converging; apex with median extension, with sparse setae; apodemes 2.3× as long as body; transfer apparatus flagelliform, ca. 3.4× as long as body of penis, subequal total length of penis; ductus ejaculatorius basally sclerotized, with indistinct bulbus.

#### Material examined.

***Holotype*** (MZB, Cole.173.057): MZB0181 (GenBank OK481869), Indonesia, C-Sulawesi, Toli-Toli, Gn. Dako, 01°03.174'N, 120°55.272'E to 01°03.389'N, 120°55.524'E, 1800–1900 m, 10-VII-2018, beaten.

#### Distribution.

C-Sulawesi Prov. (Mt Dako). Elevation ca. 1100–1200 m.

#### Biology.

On foliage in montane forest.

#### Etymology.

This epithet refers to the Corona virus (Sars-Cov2). The global pandemic led to the cancellation of field work, and a focus on this and other manuscripts. It is a noun in apposition.

#### Notes.

*Trigonopteruscorona* sp. nov. was coded as “*Trigonopterus* sp. 1235”. This species belongs to the *T.fulvicornis*-group and is related to *T.seticnemis* Riedel, from which it can be easily distinguished by the ferruginous elytra.

### 5. Trigonopterus dakoensis sp. nov.

Taxon classificationAnimaliaColeopteraCurculionidae

F66BF168-8255-5A32-B0B8-845EDC61EE95

http://zoobank.org/03951330-F918-48F9-A743-6E96205807C1

#### Diagnostic description.

**Figure 5.**
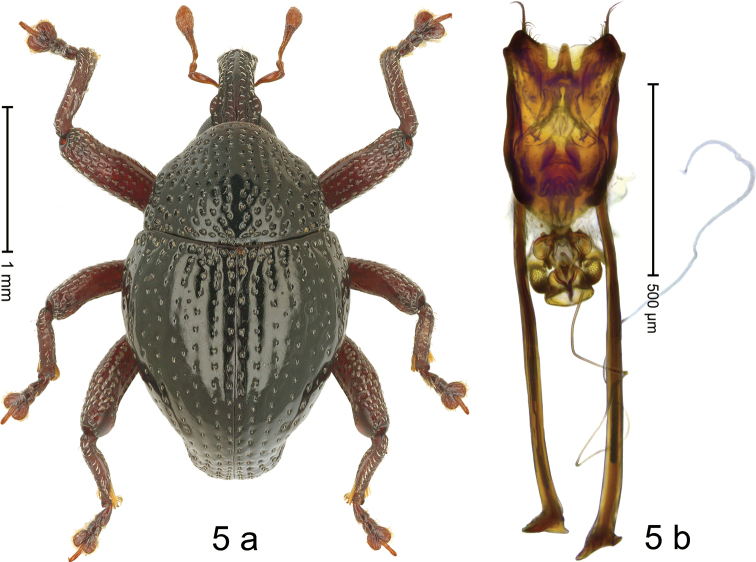
*Trigonopterusdakoensis* sp. nov., holotype **a** habitus **b** penis.

***Holotype*,** male (Fig. 5a). Length 2.58 mm. Color of antennae and legs ferruginous; remainder black. Body broad subovate; in dorsal aspect with weak constriction between pronotum and elytron, in profile dorsally convex, with very weak constriction. Rostrum dorsally with median costa and pair of submedian ridges; intervening furrows with sparse rows of suberect narrow scales; epistome short, subglabrous, with sparse setae. Pronotum densely punctate, with coarse punctures partly arranged in longitudinal rows; median line impunctate; each puncture bearing a seta; interspaces subglabrous; laterally in basal 1/3 impunctate. Elytra with striae marked by rows of punctures; basal margin bordered by transverse row of deeper, coarse punctures; interspaces subglabrous, with few small interspersed punctures; stria 8 along humerus with six coarse punctures; elytral apex subtruncate. Femora edentate; anteroventral ridge simple; anterior surface densely coarsely punctate, each puncture with recumbent scale. Metafemur with dorsoposterior edge denticulate, with silvery scales upcurved; subapically with stridulatory patch. Metatibia subapically at base of uncus with inward directed brush of yellowish setae. Abdominal ventrites 1–2 deeply concave, medially subglabrous, laterally covered with erect scales; ventrite 5 concave, at middle impunctate, surrounded by large, coarse punctures; apically with sharp, transverse ridge, forming oblique, subglabrous surface. Penis (Fig. 5b) with body in profile with constriction, its apical half swollen; apex, ventrally with acute median process, bordered by wide lobes bearing fringe of long setae; laterally with smaller pair of simple lobes; apodemes 2.4× as long as body of penis; transfer apparatus spiniform, contained by complex accessory sclerites; ductus ejaculatorius without bulbus. ***Intraspecific variation***. Length 2.38–2.58 mm. Female unknown.

#### Material examined.

***Holotype*** (MZB, Cole.173.058): MZB0064 (GenBank OK481953), Indonesia, C-Sulawesi, Toli-Toli, Gn. Dako, 01°03.512'N, 120°54.054'E, 1100 m, 13-VII-2018, sifted. ***Paratype*** (SMNK): Indonesia, C-Sulawesi, Toli-Toli, Gn. Dako: 1 ex, MZB0065 (GenBank OK481952), 01°03.531'N, 120°54.052'E, 1100 m, 13-VII-2018, sifted.

#### Distribution.

C-Sulawesi Prov. (Mt Dako). Elevation 1030–1200 m.

#### Biology.

In leaf litter of mountain forest.

#### Etymology.

This epithet is a Latinized adjective based on Mt Dako.

#### Notes.

*Trigonopterusdakoensis* sp. nov. was coded as “*Trigonopterus* sp. 1189”. The species belongs to the *T.manadensis*-group. It is related to *T.manadensis* Riedel, from which it differs by the peculiar morphology of the penis and by the longitudinal rows of coarse punctures on the pronotum. The p-distance of their *cox1* sequences is 14.5–14.8%.

### 6. Trigonopterus daun sp. nov.

Taxon classificationAnimaliaColeopteraCurculionidae

1402534F-5282-5B17-AEA5-EC77B230AEFA

http://zoobank.org/1FAC1195-2A80-44E5-9244-5AB006E919BA

#### Diagnostic description.

**Figure 6.**
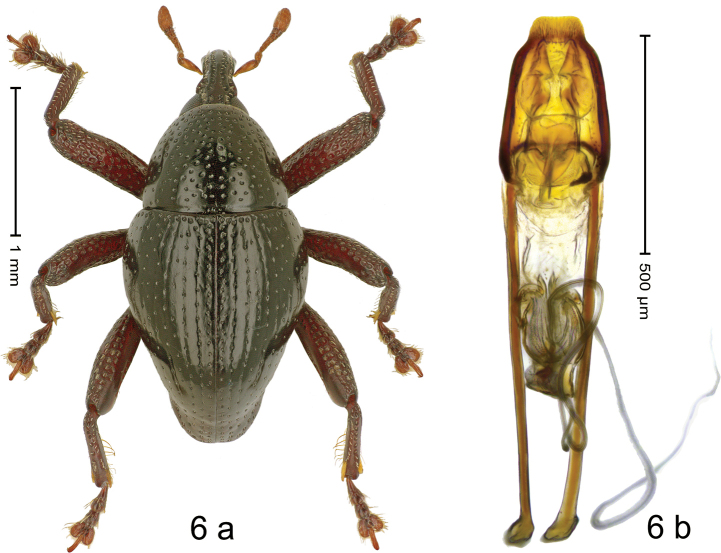
*Trigonopterusdaun* sp. nov., holotype **a** habitus **b** penis.

***Holotype*. Male** (Fig. 6a). Length 2.28 mm. Color of antennae and legs ferruginous; remainder black. Body subovate, elongate; in dorsal aspect and in profile with weak constriction between pronotum and elytron. Rostrum dorsally with median costa and pair of submedian ridges; intervening furrows with sparse rows of subrecumbent setae; epistome indistinct. Pronotum with disk densely punctate; interspaces between punctures subglabrous. Elytra with striae marked by fine hairlines and rows of small punctures; basal margin bordered row of slightly larger punctures; sutural interval subglabrous, with few interspersed punctures. Profemur with anteroventral ridge weakly crenate. Meso- and metafemur with anteroventral ridge denticulate; anterior surface of femora coarsely punctate, each puncture with narrow recumbent scale. Metafemur with dorsoposterior edge indistinct; subapically with stridulatory patch. Metatibia ventrally with sparse row of long, stiff setae. Meso- and metaventrite with plumose scales. Abdominal ventrites 1–2 concave, microreticulate, with sparse punctures and plumose scales; ventrite 5 flat, densely punctate, microreticulate. Penis (Fig. 6b) with sides of body converging, apex spatulate, truncate, without setae; ostium elongate; apodemes 2.1× as long as body of penis; transfer apparatus small, hook-shaped, directed basad in repose, without supporting sclerites; ductus ejaculatorius with indistinct bulbus. ***Intraspecific variation*.** Length 2.28–2.70 mm. Female rostrum subglabrous, with rows of small punctures. Female abdominal ventrite 5 anteriorly subglabrous, posterior half densely punctate.

#### Material examined.

***Holotype*** (MZB, Cole.173.059): MZB0099 (GenBank OK481918), Indonesia, C-Sulawesi, Toli-Toli, Gn. Dako, 01°03.697N, 120°53.991'E, 970 m, 07-VII-2018, beaten. ***Paratypes*** (MZB, SMNK): Indonesia, C-Sulawesi, Toli-Toli, Gn. Dako: 2 exx, MZB0100 (GenBank OK481917), MZB0278 (GenBank OK481778), same data as holotype; 1 ex, 01°03.967'N, 120°53.692'E to 01°03.782'N, 120°53.934'E, 830–970 m, 03-VII-2018, beaten; 2 exx, MZB0279 (GenBank OK481777), 01°03.967'N, 120°53.692'E to 01°03.782'N, 120°53.934'E, 830–970 m, 05-VII-2018, beaten; 1 ex, 01°03.782'N, 120°53.934'E, 970 m, 05-VII-2018, beaten; 2 exx, 01°03.782'N, 120°53.934'E to 01°03.574'N, 120°54.032'E, 970–1100 m, 06-VII-2018, beaten; 1 ex, MZB0200 (PCR failed), 01°03.574'N, 120°54.032'E to 01°03.181'N, 120°54.607'E, 1100–1400 m, 07-VII-2018, beaten; 1 ex, 01°03.412'N, 120°54.126'E to 01°03.241'N, 120°54.328'E, 1200–1300 m, 07-VII-2018, beaten; 1 ex, MZB0254 (GenBank OK481799), 01°02.977'N, 120°55.010'E to 01°03.782N, 120°53.934'E, 970–1740 m, 11-VII-2018.

#### Distribution.

C-Sulawesi Prov. (Mt Dako). Elevation 970–1200 m.

#### Biology.

On foliage in montane forest.

#### Etymology.

This epithet is the Indonesian word for “leaf” and a noun in apposition. It refers to the species´ lifestyle on foliage.

#### Notes.

*Trigonopterusdaun* sp. nov. was coded as “*Trigonopterus* sp. 1116” (Narakusumo et al. 2020). This species belongs to the *T.tatorensis*-group. It is closely related to *T.acutus* sp. nov., from which it can be distinguished by the erect metatibial setae, and the truncated apex of the penis. The *cox1* p-distance is 10.0–10.5%.

### 7. Trigonopterus ewok sp. nov.

Taxon classificationAnimaliaColeopteraCurculionidae

7A3B89DE-65BC-5428-B779-BD93FB7632D0

http://zoobank.org/591D85E0-1EE1-4B4F-8619-B334A05B4C91

#### Diagnostic description.

**Figure 7.**
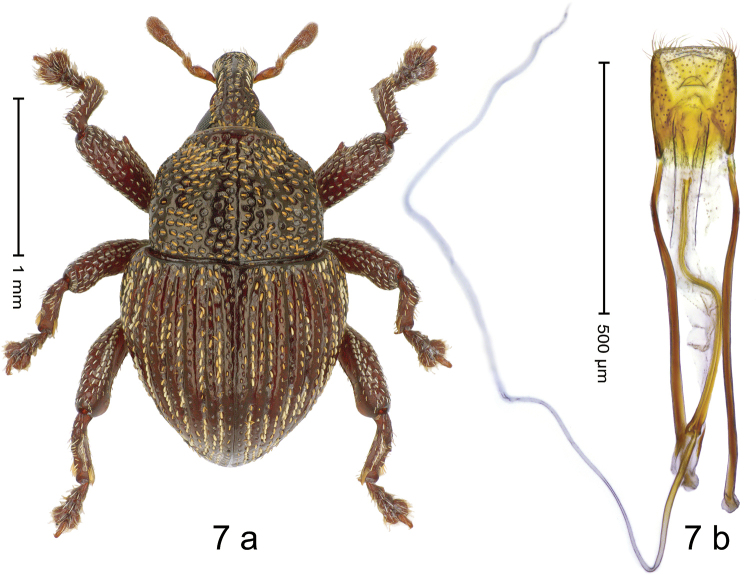
*Trigonopterusewok* sp. nov., holotype **a** habitus **b** penis.

***Holotype*. Male** (Fig. 7a). Length 2.23 mm. Color ferruginous; thorax black. Body subovate; in dorsal aspect with moderate constriction between pronotum and elytron; in profile dorsally convex. Rostrum dorsally with median and pair of submedian ridges, separated by rows of coarse punctures containing subrecumbent yellow scales; epistome subglabrous, with sparse setae and minute punctures, posteriorly with transverse ridge. Pronotum with indistinct lateral edges subparallel to weak subapical constriction; disk with dense, partly confluent coarse punctures, with recumbent yellow scales; with subglabrous midline. Elytra with striae deeply impressed, containing rows of yellow or white scales; intervals costate, with rows of punctures and yellowish recumbent scales. Femora dentate, with acute tooth; anteroventral ridge weakly crenulate; dorsal and anterior surface with sparse white scales. Metafemur with dorsoposterior edge indistinct, weakly denticulate; subapically with extended stridulatory patch; dorsally with subrecumbent silvery scales. Dorsoposterior edge of tibiae subbasally angulate, denticulate. Abdominal ventrites 1–2 weakly concave, with dense coarse punctures, with sparse piliform scales; ventrite 5 with weak impression, densely punctate, with sparse suberect setae. Penis (Fig. 7b) with sides of body subparallel, apex subtruncate, with dense setae; apodemes 3.3× as long as body of penis; transfer apparatus flagelliform; ductus ejaculatorius basally sclerotized, with indistinct bulbus. ***Intraspecific variation***. Length 1.93–2.23 mm. Epistome of female rostrum posteriorly without transverse ridge. Female pronotum with yellow scales concentrated in sublateral bands. Elytra with scaling more or less extensive. Female abdominal ventrites 1–2 flat.

#### Material examined.

***Holotype*** (MZB, Cole.173.060): MZB0129 (GenBank OK481905), Indonesia, C-Sulawesi, Tojo Una-Una, Matako, Gn. Pompangeo, 01°35.215'S, 120°55.560'E to 01°35.079'S, 120°55.49'E, 1900 m, 28-II-2020, sifted. ***Paratypes*** (MZB, SMNK): Indonesia, C-Sulawesi, Tojo Una-Una, Matako, Gn. Pompangeo: 1 ex, MZB0128 (GenBank OK481906), 01°35.235'S, 120°55.679'E to 01°35.258'S, 120°55.588'E, 1900 m, 27-II-2020, sifted; 2 exx, MZB0130 (GenBank OK481904), 01°35.197'S, 120°55.658'E to 01°35.127'S, 120°55.622'E, 2000 m, 01-III-2020, sifted.

#### Distribution.

C-Sulawesi Prov. (Mt Pompangeo). Elevation ca. 1900–2000 m.

#### Biology.

In leaf litter of montane forest.

#### Etymology.

This epithet is a noun in apposition based on the fictional character of small bear-like creatures from Star Wars VI movie.

#### Notes.

*Trigonopterusewok* sp. nov. was coded as “*Trigonopterus* sp. 1188” (Narakusumo et al. 2020). This species belongs to the *T.impressicollis*-group. It is closely related to *T.impressicollis* Riedel, which differs by the absence of longitudinal impressions on the pronotum, shorter setae on the apex of penis and 17.5–17.7% *cox1* p-distance.

### 8. Trigonopterus gundala sp. nov.

Taxon classificationAnimaliaColeopteraCurculionidae

8C034FFE-3206-5F6F-904B-1327D92CF6A4

http://zoobank.org/87D8C696-B01C-4E94-A1A8-28238FDC716F

#### Diagnostic description.

**Figure 8.**
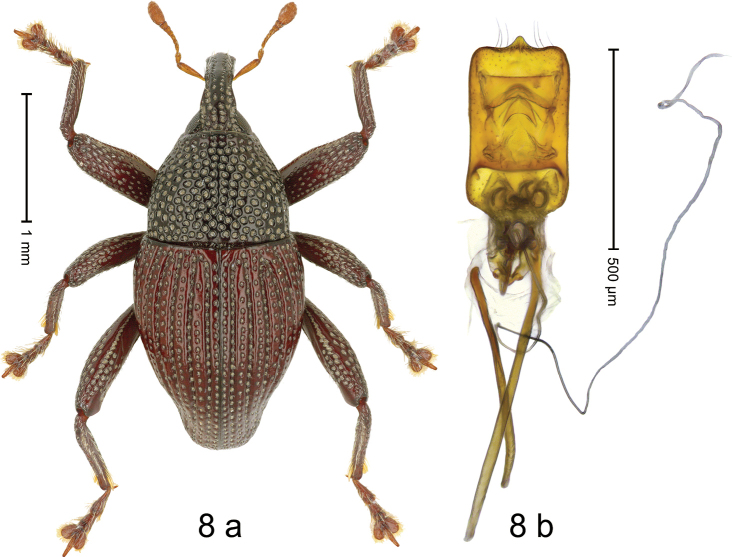
*Trigonopterusgundala* sp. nov., holotype **a** habitus **b** penis.

***Holotype*.** Male (Fig. 8a). Length 2.75 mm. Color of antennae, elytra and legs ferruginous; head and thorax black. Body elongate subovate; in dorsal aspect and in profile with distinct constriction between pronotum and elytron. Rostrum dorsally with subglabrous median costa and pair of submedian ridges; intervening furrows with coarse punctures and subrecumbent setae; apical 1/3 subglabrous, with few punctures and with sparse suberect setae. Pronotum with disk reticulate-punctate; ridges between punctures subglabrous; laterally in basal 1/3 with sparser punctures. Elytra with striae deeply impressed; intervals costate, each with one row of greyish punctures containing minute seta; suture at middle weakly carinate. Femora edentate; with distinct anteroventral and posteroventral ridges simple; anterior surface densely coarsely punctate, each puncture with recumbent scale. Metafemur dorsally with row of confluent punctures, bordered by subglabrous line; subapically with stridulatory patch. Metatibia slender, with sparse suberect setae. Abdominal ventrites 1 and 2 flat with coarse punctures; ventrite 5, weakly concave towards apex, with dense small punctures. Penis (Fig. 8b) with sides of body subparallel; apex with median angulate extension and sparse long setae; apodemes 1.5× as long as body of penis; transfer apparatus spiniform, directed basad in repose; ductus ejaculatorius without bulbus. ***Intraspecific variation***. Length 2.55–2.76 mm. Female rostrum slender, subglabrous. Female ventrite 1 and 2 flat with dense small punctures. Female ventrite 5 flat.

#### Material examined.

***Holotype*** (MZB, Cole.173.061): MZB0079 (GenBank OK481938), Indonesia, C-Sulawesi, Toli-Toli, Gn. Dako, 01°03.389'N, 120°55.524'E to 01°03.567'N, 120°56.032'E, 1900–2200 m, 10-VII-2018, beaten. ***Paratypes*** (MZB, SMNK): Indonesia, C-Sulawesi, Toli-Toli, Gn. Dako: 2 exx, MZB0080 (GenBank OK481937), MZB0190 (GenBank OK481860), same data as holotype; 4 exx, MZB0082 (GenBank OK481935), MZB0186 (GenBank OK481864), MZB0187 (GenBank OK481863), MZB0188 (GenBank OK481862), 01°03.174N, 120°55.272'E to 01°03.389N, 120°55.524'E, 1800–1900 m, 10-VII-2018, beaten.

#### Distribution.

C-Sulawesi Prov. (Mt Dako). Elevation ca. 1900–2200 m.

#### Biology.

On foliage in montane forest.

#### Etymology.

This epithet is a noun in apposition based on the fictional character of Indonesian comic superhero “Gundala, Son of Thunder”. The black and ferruginous colors of this species resemble Gundala’s movie costume.

#### Notes.

*Trigonopterusgundala* sp. nov. was coded as “*Trigonopterus* sp. 1194”. It belongs to the *T.satyrus*-group and is closely related to *T.mons* sp. nov., but differs by the deeply striate elytra and a 8.2–8.7% *cox1* p-distance.

### 9. Trigonopterus hoppla sp. nov.

Taxon classificationAnimaliaColeopteraCurculionidae

57CC1922-600D-5A9E-9DFA-B534D52D06A6

http://zoobank.org/80388645-F13D-4FFC-9AB2-DC34EC40DFFB

#### Diagnostic description.

**Figure 9.**
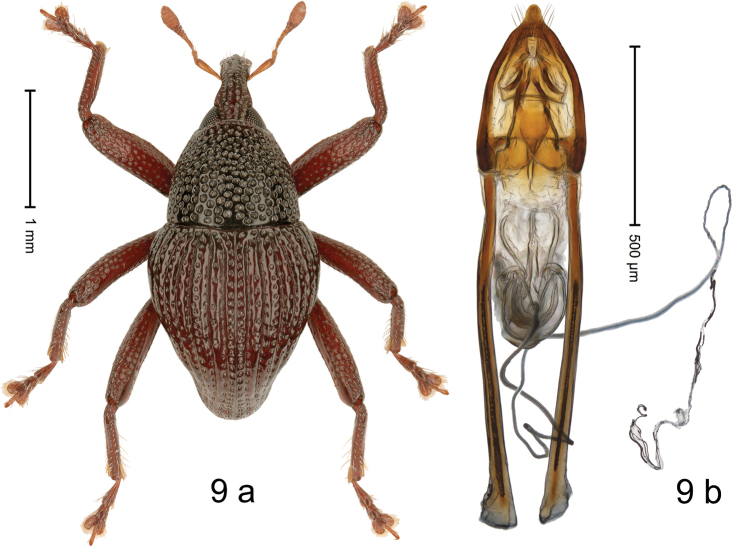
*Trigonopterushoppla* sp. nov., holotype **a** habitus **b** penis.

***Holotype*. Male** (Fig. 9a). Length 2.75 mm. Color of antennae and legs ferruginous; elytra dark ferruginous, almost black; remainder black. Body subovate; in dorsal aspect and in profile with weak constriction between pronotum and elytron. Rostrum dorsally with median costa and pair of submedian ridges; intervening furrows with rows of coarse punctures, each puncture containing one subrecumbent seta; epistome indistinct. Pronotum with disk densely punctate-reticulate with coarse punctures. Elytra with striae deeply impressed; intervals costate, each with one row of punctures. Profemur with anteroventral ridge simple. Meso- and metafemur with anteroventral ridge crenate; anterior surface of femora coarsely punctate, each puncture with narrow recumbent scale. Metafemur with dorsoposterior edge indistinct; subapically with stridulatory patch. Metatibia ventrally with few long, stiff setae. Meso- and metaventrite with sparse plumose scales. Abdominal ventrites 1–2 concave, markedly microreticulate, with coarse punctures and sparse plumose scales; ventrite 5 flat, with shallow impression, densely punctate, microreticulate. Penis (Fig. 9b) with sides of body converging to pointed apex; ostium with complex sclerites; apodemes 2.4× as long as body of penis; transfer apparatus spiniform, without supporting sclerites; ductus ejaculatorius with indistinct bulbus. ***Intraspecific variation***. Length 2.69–2.75 mm. Sculpture of paratype much less distinct; moderately to sparsely punctate, elytral striae weakly impressed.

#### Material examined.

***Holotype*** (MZB, Cole.173.062): MZB0201 (GenBank OK481850), Indonesia, C-Sulawesi, Toli-Toli, Gn. Dako, 01°02.977'N, 120°55.009'E to 01°03.210'N, 120°55.297'E, 1700–1800 m, 08–10-VII-2018, beaten. ***Paratype*** (SMNK): Indonesia, C-Sulawesi, Toli-Toli, Gn. Dako: 1 ex, MZB0277 (GenBank OK481779), 01°03.412'N, 120°54.126'E to 01°03.241'N, 120°54.328'E, 1200–1300 m, 07-VII-2018.

#### Distribution.

C-Sulawesi Prov. (Mt Dako). Elevation 1300–1700 m.

#### Biology.

On foliage in montane forest.

#### Etymology.

This epithet is based on the German word “Hoppla”, an exclamation of surprise, comparable to the English “whoops”. It is to be treated as a noun in apposition.

#### Notes.

*Trigonopterushoppla* sp. nov. was coded as “*Trigonopterus* sp. 1232”. This species belongs to the *T.tatorensis*-group. It is closely related to *T.daun* sp. nov., from which it can be distinguished by the pointed apex of the penis and a *cox1* p-distance of 7.8%. The marked difference in sculpture between holotype and the single paratype is remarkable, and would usually indicate a separate species. However, genital morphology and *cox1* sequence of both specimens are almost identical, so either the coarse sculpture of the holotype, or the smooth sculpture of the paratype may be an aberration. Additional specimens are needed to clarify this matter.

### 10. Trigonopterus kakimerah sp. nov.

Taxon classificationAnimaliaColeopteraCurculionidae

716CB4EC-0232-5648-8E08-3911426282BA

http://zoobank.org/4DA046DE-E058-4E96-BF43-DCD6FF75047E

#### Diagnostic description.

**Figure 10.**
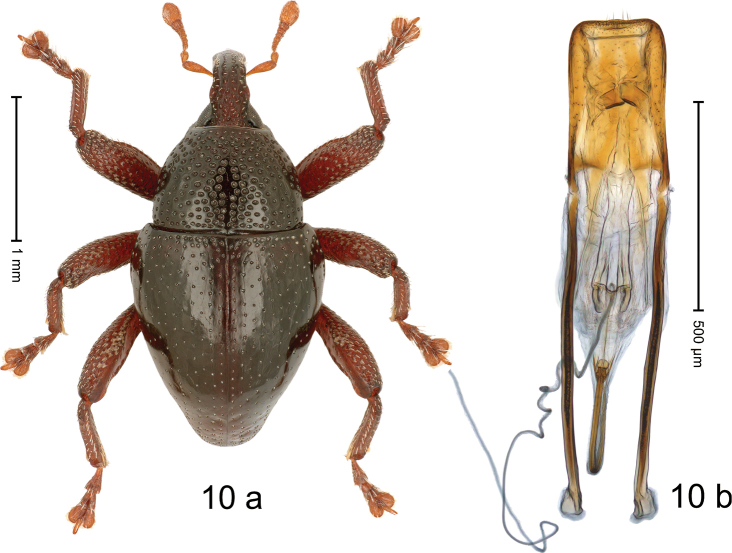
*Trigonopteruskakimerah* sp. nov., holotype **a** habitus **b** penis.

***Holotype*. Male** (Fig. 10a). Length 2.30 mm. Color of antennae and legs ferruginous, remainder black. Body subovate; in dorsal aspect with weak constriction between pronotum and elytron; in profile dorsally convex. Rostrum punctate-rugose, in basal half with median and pair of submedian ridges, in apical half punctate. Pronotum with disk densely punctate, interspace subglabrous; median line impunctate. Elytra irregularly punctate with small punctures; interspaces subglabrous; along basal margin with denser punctures; stria 8 along humerus with five coarse punctures externally bordered by weak costa. Femora with anteroventral ridge crenulate, in metafemur shortened, forming blunt tooth. Metafemur dorsally with sparse slender scales; without distinct dorsoposterior edge; subapically with stridulatory patch. Abdominal ventrites 1–2 concave, subglabrous, sparsely punctate, microreticulate; ventrite 5 with shallow median impression, coarsely punctate, microreticulate. Penis (Fig. 10b) with sides of body subparallel to rounded apex; apodemes 2.0× as long as body of penis; endophallus with pair of elongate sclerites; transfer apparatus flagelliform, ca. 1.2× as long as body of penis, coiled up in apical portion of endophallus; ductus ejaculatorius without bulbus. ***Intraspecific variation*.** Length 2.28–2.35 mm. Female rostrum slender, dorsally subglabrous, with submedian row of punctures. Female ventrite 5 without median impression.

#### Material examined.

***Holotype*** (MZB, Cole.173.063): MZB0207 (GenBank OK481846), Indonesia, C-Sulawesi, Tojo Una-Una, Matako, Gn. Pompangeo, 01°35.603'S, 120°55.437'E to 01°35.406'S, 120°55.547'E, 1800 m, 29-II-2020, beaten. ***Paratype*** (MZB, SMNK): Indonesia, C-Sulawesi, Tojo Una-Una, Matako, Gn. Pompangeo: 1 ex, MZB0208 (GenBank OK481845), 01°35.264'S, 120°55.588'E to 01°35.339'S, 120°55.599'E, 1900 m, 29-II-2020, beaten; 2 exx, MZB0209 (GenBank OK481844), MZB0210 (GenBank OK481843), 01°35.264'S, 120°55.588'E to 01°35.339'S, 120°55.599'E, 1900 m, 26–27-II-2020, beaten; 1 ex, MZB0154 (GenBank OK481884), 01°35.197'S, 120°55.658'E to 01°35.154'S, 120°55.507'E, 1900 m, 28-II-2020.

#### Distribution.

C-Sulawesi Prov. (Mt Pompangeo). Elevation 1800–1900 m.

#### Biology.

On foliage in montane forest.

#### Etymology.

This epithet is the Indonesian term for “red legs”. It is a noun in apposition.

#### Notes.

*Trigonopteruskakimerah* sp. nov. was coded as “*Trigonopterus* sp. 1202”. This species may belong to the *T.fulvicornis*-group. The *cox1* p-distance to other known species is above 14%.

### 11. Trigonopterus katopasensis sp. nov.

Taxon classificationAnimaliaColeopteraCurculionidae

03341C24-BC4B-5CD2-B2C8-AF8D02F482E5

http://zoobank.org/EFE5C4A8-8D53-4302-A54C-9E57C4165620

#### Diagnostic description.

**Figure 11.**
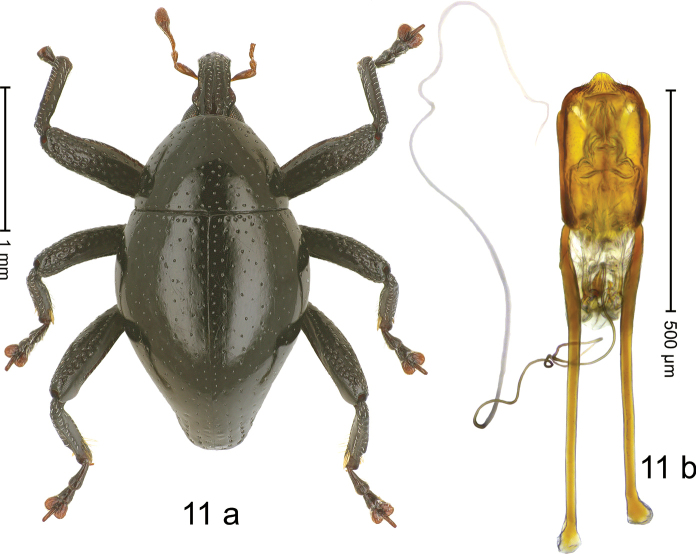
*Trigonopteruskatopasaensis* sp. nov., holotype **a** habitus **b** penis.

***Holotype*. Male** (Fig. 11a). Length 2.35 mm. Color of antennae and apical tarsomeres ferruginous; remainder black. Body subovate; in dorsal aspect with weak constriction between pronotum and elytron; in profile dorsally convex. Rostrum dorsally with median and pair of submedian carinae; intervening furrows with sparse rows of subrecumbent setae; epistome indistinct, subglabrous, subapically with sparse suberect setae. Pronotum with disk sparsely punctate with small punctures; interspaces subglabrous. Elytra sparsely punctate with irregular, minute punctures; interspaces subglabrous; striae indistinct; along basal margin with slightly denser row. Femora with anteroventral ridge crenulate; anterior surface microreticulate, dorsally and especially subapically coarsely punctate. Metafemur with dorsoposterior edge indistinct; subapically with stridulatory patch. Metatibia in apical half ventrally with sparse row of long, stiff setae; dorsal contour in apical third weakly emarginate. Abdominal ventrites 1–2 concave, subglabrous; ventrite 5 microreticulate, with subquadrate pit, bordered by subparallel lateral ridges. Penis (Fig. 11b) with sides of body subparallel; apex symmetrical, with angulate extension; with few setae; apodemes 2.2× as long as body of penis; transfer apparatus complex; ductus ejaculatorius with indistinct bulbus. ***Intraspecific variation*.** Length 1.98–2.68 mm. Female rostrum subglabrous, with distinct furrows, costae indistinct. Female abdominal ventrites 1–2 less concave; ventrite 5 flat.

#### Material examined.

***Holotype*** (MZB, Cole.173.064): MZB0108 (GenBank OK481912) Indonesia, C-Sulawesi, Tojo Una-Una, Ulubongka, Mire, Gn. Katopasa, 01°11.31'S, 121°27.348'E, 1380 m, 23–24-VII-2017, beaten. ***Paratype*** (MZB, SMNK): 27 exx, MZB0109 (GenBank OK481911) same data as holotype.

#### Distribution.

C-Sulawesi Prov. (Mt Katopasa). Elevation ca. 1380 m.

#### Biology.

On foliage in montane forest.

#### Etymology.

This epithet is a Latinized adjective based on Mt Katopasa.

#### Notes.

*Trigonopteruskatopasensis* sp. nov. was coded as “*Trigonopterus* sp. 1190”. This species presumably belongs to the *T.barbipes*-group. From *T.barbipes* Riedel it differs by less distinct body sculpture, the structure of the penis, and a 18.5% *cox1* p-distance.

### 12. Trigonopterus matakensis sp. nov.

Taxon classificationAnimaliaColeopteraCurculionidae

7A5459B2-ACD3-55D0-B1A9-D92481A009D9

http://zoobank.org/8F5E17E6-7640-4C26-81EF-B6CF87664D13

#### Diagnostic description.

**Figure 12.**
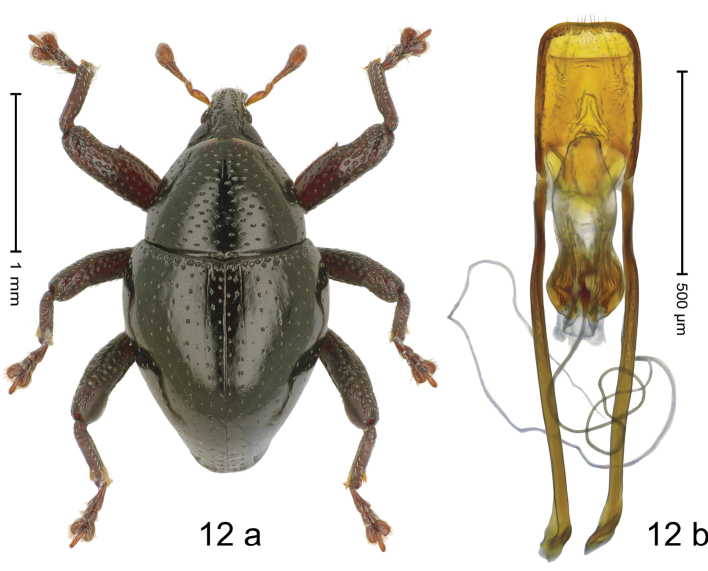
*Trigonopterusmatakensis* sp. nov., holotype **a** habitus **b** penis.

***Holotype*. Male** (Fig. 12a). Length 2.13 mm. Color of antennae ferruginous, legs dark ferruginous, remainder black. Body subovate; in dorsal aspect with weak constriction between pronotum and elytron; in profile dorsally convex, with very weak constriction between pronotum and elytron. Rostrum dorsally with median costa and with pair of irregular submedian ridges; intervening furrows with sparse rows of suberect setae, converging towards apex; epistome indistinct, subglabrous, with sparse erected setae. Pronotum with ovate punctures of transverse orientation; interspaces subglabrous. Elytra with striae marked by rows of small punctures; sutural interval with additional row, other intervals subglabrous, with sparse minute punctures; basal margin with transverse row of somewhat larger punctures. Femora dentate with small tooth; anterior surface punctate, microreticulate, each puncture with short recumbent seta. Meso- and metafemur with anteroventral ridge crenate. Metafemur subapically with stridulatory patch. Abdominal ventrites 1–2 concave, microreticulate, with sparse punctures; ventrite 5 punctate, microreticulate, with sparse setae, at middle with distinct impression. Penis (Fig. 12b) with body subparallel to rounded apex, with few short setae; apodemes 2.6× as long as body of penis; transfer apparatus spiniform, pointing apicad, held by subrotund sclerite; ductus ejaculatorius without bulbus. ***Intraspecific variation***. Length 1.98–2.43 mm. Female rostrum smooth, subglabrous, with rows of small punctures. Female ventrite 5 flat, densely punctate, microreticulate.

#### Material examined.

***Holotype*** (MZB, Cole.173.065): MZB0159 (GenBank OK481879), Indonesia, C-Sulawesi, Tojo Una-Una, Matako, Gn. Pompangeo, 01°35.603'S, 120°55.437'E to 01°35.406'S, 120°55.547'E, 1800 m, 29-II-2020, beaten. ***Paratypes*** (MZB, SMNK): Indonesia, C-Sulawesi, Tojo Una-Una, Matako, Gn. Pompangeo: 1 ex, MZB0148 (GenBank OK481889), 01°35.215'S, 120°55.560'E to 01°35.079'S, 120°55.49'E, 1900 m, 28-II-2020, beaten; 1 ex, MZB0149 (GenBank OK481888), 01°35.603'S, 120°55.437'E to 01°35.406'S, 120°55.547'E, 1800 m, 29-II-2020, beaten; 4 exx, MZB0223 (GenBank OK481830), MZB0224 (GenBank OK481829), MZB0225 (GenBank OK481828), MZB0226 (GenBank OK481827), 01°35.359'S, 120°55.643'E to 01°35.581'S, 120°55.385'E, 1800 m, 29-II-2020, beaten; 2 exx, MZB0156 (GenBank OK481882), MZB0157 (GenBank OK481881), 01°35.359'S, 120°55.643'E to 01°35.581'S, 120°55.385'E, 1900 m, 29-II-2020, sifted; 3 exx, MZB0158 (GenBank OK481880), MZB0234 (GenBank OK481819), MZB0235 (GenBank OK481818), 01°35.235'S, 120°55.679'E to 01°35.258'S, 120°55.588'E, 1900 m, 26–27-II-2020, beaten; 2 exx, MZB0166 (GenBank OK481872), MZB0167 (GenBank OK481871), 01°35.603'S, 120°55.437'E to 01°35.406'S, 120°55.547'E, 1800 m, 29-II-2020, beaten; 5 exx, MZB0168 (GenBank OK481870), MZB0230 (GenBank OK481823), MZB0231 (GenBank OK481822), MZB0232 (GenBank OK481821), MZB0233 (GenBank OK481820), 01°35.197'S, 120°55.658'E to 01°35.154'S, 120°55.507'E, 1900 m, 28-II-2020, beaten; 10 exx, MZB0150 (GenBank OK481887), MZB0155 (GenBank OK481883), MZB0217 (GenBank OK481836), MZB0218 (GenBank OK481835), MZB0219 (GenBank OK481834), MZB0220 (GenBank OK481833), MZB0221 (GenBank OK481832), MZB0222 (GenBank OK481831), MZB0227 (GenBank OK481826), MZB0228 (GenBank OK481825), 01°35.197'S, 120°55.658'E to 01°35.127'S, 120°55.622'E, 2000 m, 01-III-2020, beaten.

#### Distribution.

C-Sulawesi Prov. (Mt Pompangeo). Elevation 1800–2000 m.

#### Biology.

On foliage and in leaf litter in montane forests.

#### Etymology.

This epithet is a Latinized adjective based on Matako village.

#### Notes.

*Trigonopterusmatakensis* sp. nov. was coded as “*Trigonopterus* sp. 1197”. This species belongs to the *T.ovalipunctatus*-group. It is closely related to *T.ovalipunctatus* Riedel, but differs by the subrotund shape of the supporting sclerites of the transfer apparatus and a 9.6–10.3% *cox1* p-distance.

### 13. Trigonopterus moduai sp. nov.

Taxon classificationAnimaliaColeopteraCurculionidae

B717B1AC-BCD5-542E-988B-277FC88192CC

http://zoobank.org/3A311395-FA62-492C-85CF-C5DFE56F1ED1

#### Diagnostic description.

**Figure 13.**
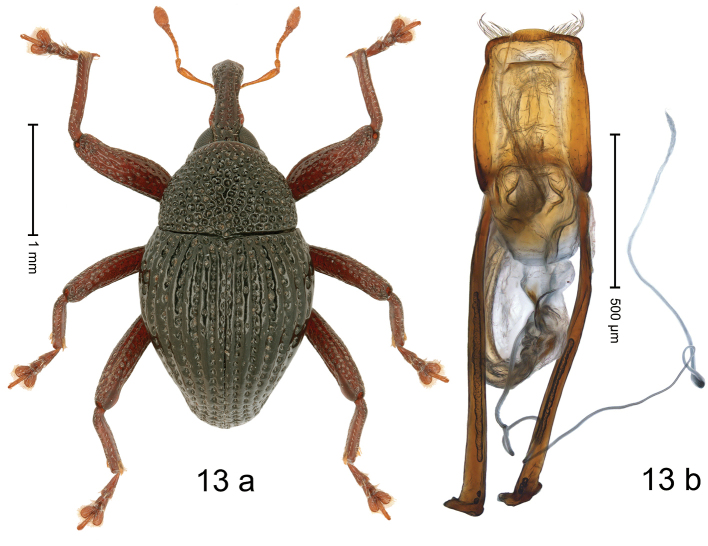
*Trigonopterusmoduai* sp. nov., holotype **a** habitus **b** penis.

***Holotype*. Male** (Fig. 13a). Length 2.80 mm. Color of antennae and legs ferruginous; remainder black. Body subovate; in profile with weak constriction between pronotum and elytron. Rostrum at middle with constriction; dorsally coarsely punctate-rugose, in basal half with sublateral furrows containing rows of suberect setae; epistome indistinct, subglabrous with sparse setae. Pronotum with disk coarsely punctate-reticulate; interspaces between punctures subglabrous; each puncture with one minute seta. Elytra with striae marked by deep punctures each with one minute seta; intervals costate, subglabrous, with few interspersed punctures. Femora edentate; anteroventral ridges simple. Metafemur with dorsoposterior edge indistinct; subapically with stridulatory patch. Abdominal ventrites 1–2 concave, microreticulate, with coarse punctures; ventrite 5 with broad, shallow impression, microreticulate, with sparse small punctures. Penis (Fig. 13b) with sides of body subparallel, weakly converging, subapically with shallow constriction; apex setose, with median rounded extension; basal orifice ventrally with brace; apodemes 2.2× as long as body of penis; transfer apparatus flagelliform, looping S-shaped apicad, its tip emerging from apical orifice, ca. 3.5× as long as body of penis; ductus ejaculatorius without bulbus. ***Intraspecific variation***. Length 2.58–2.80 mm. Female rostrum more slender, punctures smaller. Female ventrites 1–2 almost flat.

#### Material examined.

***Holotype*** (MZB, Cole.173.066): MZB0072 (GenBank OK481945), Indonesia, C-Sulawesi, Toli-Toli, Gn. Dako, 01°02.977'N, 120°55.010'E to 01°03.210'N, 120°55.297'E, 1700–1800 m, 08–10-VII-2018, beaten. ***Paratypes*** (MZB, SMNK): Indonesia, C-Sulawesi, Toli-Toli, Gn. Dako: 14 exx, MZB0071 (GenBank OK481946), MZB0073 (GenBank OK481944), MZB0165 (GenBank OK481873), same data as holotype; 1 ex, MZB0070 (GenBank FD03047006), 01°03.181'N, 120°54.607'E to 01°02.977'N, 120°55.001'E, 1400–1750 m, 07-VII-2018.

#### Distribution.

C-Sulawesi Prov. (Mt Dako). Elevation ca. 1700–1800 m.

#### Biology.

On foliage in montane forest.

#### Etymology.

This epithet is a noun in apposition based on “Moduai”, a folk dance of people from Toli-Toli Regency.

#### Notes.

*Trigonopterusmoduai*, sp. nov. was coded as “*Trigonopterus* sp. 1201” and belongs to the *T.arachnobas*-group. It is very close to *T.paramoduai* sp. nov. (3.20–4.11% *cox1* p-distance) but can be distinguished by the darker elytral color, a slightly shorter rostrum, and the much longer flagellum of the male genital.

### 14. Trigonopterus mons sp. nov.

Taxon classificationAnimaliaColeopteraCurculionidae

D9D4007B-3CE4-5383-A652-0A558CB49F24

http://zoobank.org/CDF8BEDA-26ED-4254-9838-EA6285D263C1

#### Diagnostic description.

**Figure 14.**
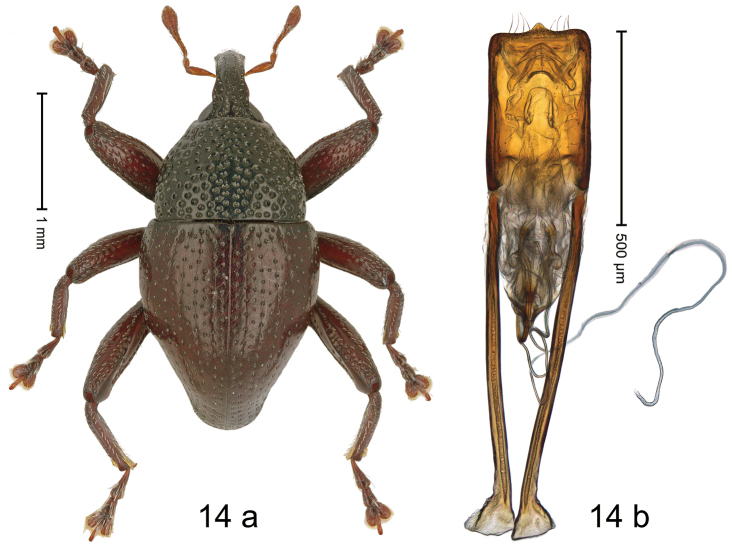
*Trigonopterusmons* sp. nov., holotype **a** habitus **b** penis.

***Holotype*. Male** (Fig. 14a). Length 2.81 mm. Color of antennae and legs ferruginous; elytra dark ferruginous; remainder black. Body subovate; in dorsal aspect with weak constriction between pronotum and elytron; in profile dorsally convex. Rostrum dorsally with flattened median costa and pair of submedian costae; intervening furrows with coarse punctures and suberect setae; apical 1/3 subglabrous, with suberect setae; epistome indistinct. Pronotum with disk densely punctate with coarse punctures; interspaces between punctures subglabrous; laterally in basal 1/3 subglabrous. Elytra with striae marked by punctures and fine hairlines; intervals subglabrous, with interspersed punctures. Femora edentate; anteroventral ridges simple. Metafemur with dorsoposterior edge simple; subapically with stridulatory patch. Abdominal ventrites 1–2 concave, medially subglabrous, laterally microreticulate, with coarse punctures; ventrite 5 with subquadrate pit, subglabrous, weakly microreticulate, subapically with short median carina; laterally punctate. Penis (Fig. 14b) with sides of body subparallel; apex subtruncate, with median triangular extension, with sparse long setae; apodemes 2.4× as long as body of penis; transfer apparatus flagelliform, looping apicad; support structures elongate lyriform; ductus ejaculatorius without bulbus. ***Intraspecific variation***. Length 2.44–3.13 mm. Female rostrum more slender, subglabrous, with rows of punctures. Female ventrites 1–2 weakly concave. Female ventrite 5 flat, subglabrous, with sparse punctures.

#### Material examined.

***Holotype*** (MZB, Cole.173.067): Indonesia, C-Sulawesi, Toli-Toli, Gn. Dako, MZB0162 (GenBank OK481876), 01°03.181'N, 120°54.607'E to 01°02.977'N, 120°55.001'E, 1400–1750 m, 07-VII-2018, beaten. ***Paratypes*** (MZB, SMNK): Indonesia, C-Sulawesi, Toli-Toli, Gn. Dako: 3 exx, 01°03.782'N, 120°53.934'E to 01°02.977'N, 120°55.001'E, 1250–1750 m, 11-VII-2018, beaten; 13 exx, MZB0078 (GenBank OK481939), 01°03.389'N, 120°55.524'E to 01°03.567'N, 120°56.032'E, 1900–2200 m, 10-VII-2018, beaten; 16 exx, MZB0081 (GenBank OK481936), MZB0189 (GenBank OK481861), 01°03.174'N, 120°55.272'E to 01°03.389'N, 120°55.524'E, 1800–1900 m, 10-VII-2018, beaten; 16 exx, MZB0083 (GenBank OK481934), MZB0084 (GenBank OK481933), 01°02.977'N, 120°55.001'E to 01°03.210'N, 120°55.297'E, 1700–1800 m, 10-VII-2018, beaten; 5 exx, MZB0163 (GenBank OK481875), same as holotype.

#### Distribution.

C-Sulawesi Prov. (Mt Dako). Elevation 1700–1900 m.

#### Biology.

On foliage in montane forest.

#### Etymology.

This epithet is the Latin noun *mons* (mountain) in apposition.

#### Notes.

*Trigonopterusmons* sp. nov. was coded as “*Trigonopterus* sp. 1195”. It belongs to the *T.satyrus*-group and is closely related to *T.gundala* sp. nov., which differs by the deeply striate elytra and a 8.2–8.7% *cox1* p-distance.

### 15. Trigonopterus paramoduai sp. nov.

Taxon classificationAnimaliaColeopteraCurculionidae

9A6E8850-5A3F-54E5-A0B0-726388FBECFB

http://zoobank.org/639E9A24-FC23-49D7-B692-67D42B8F423B

#### Diagnostic description.

**Figure 15.**
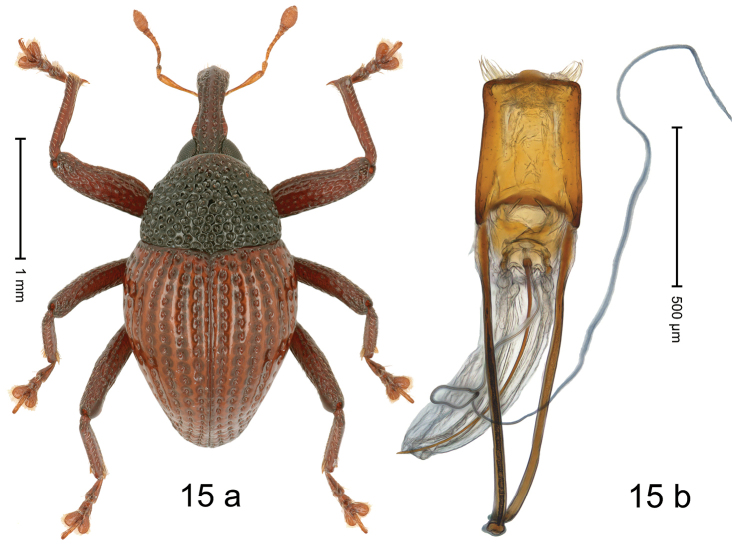
*Trigonopterusparamoduai* sp. nov., holotype **a** habitus **b** penis.

***Holotype*. Male** (Fig. 15a). Length 2.56 mm. Color of antennae, legs and elytra ferruginous; remainder black. Body subovate; in profile with weak constriction between pronotum and elytron. Rostrum at middle with constriction; dorsally coarsely punctate-rugose, punctures containing rows of suberect setae; epistome indistinct, subglabrous with sparse setae. Pronotum with disk coarsely punctate-reticulate; interspaces between punctures subglabrous; each puncture with one minute seta. Elytra with striae marked by deep punctures each with one minute seta; intervals weakly costate, subglabrous. Femora edentate; anteroventral ridges simple. Metafemur with dorsoposterior edge indistinct; subapically with stridulatory patch. Abdominal ventrites 1–2 concave, with scattered coarse punctures; ventrite 5 with broad shallow impression, with sparse small punctures. Penis (Fig. 15b) with sides of body weakly concave, converging; apex setose, with distinct median extension; basal orifice ventrally with brace; apodemes 2.4× as long as body of penis; transfer apparatus flagelliform, curved, pointing basad, ca. 2.0× as long as body of penis; ductus ejaculatorius without bulbus. ***Intraspecific variation***. Length 2.56–2.72 mm. Female rostrum more slender, punctures smaller. Female ventrites 1–2 almost flat.

#### Material examined.

***Holotype*** (MZB, Cole.173.068): MZB0183 (GenBank OK481867), Indonesia, C-Sulawesi, Toli-Toli, Gn. Dako, 01°03.389'N, 120°55.524'E to 01°03.567'N, 120°56.032'E, 1900–2200 m, 10-VII-2018, beaten. ***Paratypes*** (MZB, SMNK): Indonesia, C-Sulawesi, Toli-Toli, Gn. Dako: 3 exx, MZB0182 (GenBank OK481868), MZB0184 (GenBank OK481866), MZB0185 (GenBank OK481865), same as holotype; 2 exx, MZB0074 (GenBank OK481943), MZB0075 (GenBank OK481942), 01°03.174'N, 120°55.272'E to 01°03.389'N, 120°55.524'E, 1800–1900 m, 10-VII-2018, beaten; 2 exx, MZB0076 (GenBank OK481941), MZB0077 (GenBank OK481940), 01°03.389'N, 120°55.524'E to 01°03.567'N, 120°56.032'E, 1900–2200 m, 10-VII-2018, beaten.

#### Distribution.

C-Sulawesi Prov. (Mt Dako). Elevation 1900–2200 m.

#### Biology.

On foliage in montane forest.

#### Etymology.

This epithet is based on the combination of the Greek prefix *para*- (next to; near by) and the sibling species *Trigonopterusmoduai*, sp. nov..

#### Notes.

*Trigonopterusparamoduai* sp. nov. was coded as “*Trigonopterus* sp. 1233” and belongs to the *T.arachnobas*-group. It is very close to *T.moduai* sp. nov. (3.20–4.11% *cox1* p-distance) from which it can be distinguished by the ferruginous elytral color, a slightly longer rostrum, and the shorter flagellum of the male genital.

### 16. Trigonopterus pomberimbensis sp. nov.

Taxon classificationAnimaliaColeopteraCurculionidae

3EAD9047-4DF3-56C4-B6D1-3C4B0FE2D432

http://zoobank.org/24840861-FE15-45AE-9FB7-9D33AEF4325B

#### Diagnostic description.

**Figure 16.**
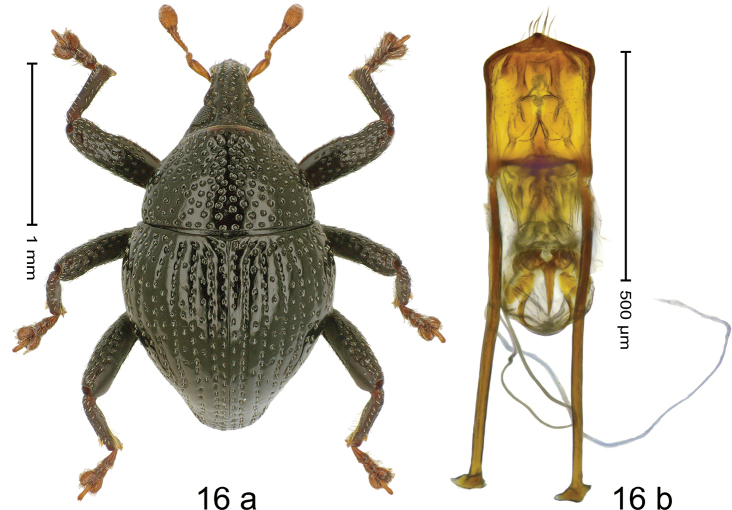
*Trigonopteruspomberimbensis* sp. nov., holotype **a** habitus **b** penis.

***Holotype*. Male** (Fig. 16a). Length 1.98 mm. Color of antennae and tarsi ferruginous; remainder black. Body subovate; in dorsal aspect and in profile with weak constriction between pronotum and elytron. Rostrum dorsally with median and pair of submedian ridges; intervening furrows with sparse rows of suberect setae; epistome indistinct, subglabrous. Pronotum with disk densely punctate; interspaces between punctures subglabrous, subequal or smaller than punctures´ diameter; laterally punctures coarse. Elytra with striae marked by weakly impressed rows of punctures; intervals subglabrous, with interspersed punctures. Femora with anteroventral ridge weakly crenate, ending with small acute tooth; anterior surface coarsely punctate, microreticulate. Metafemur with dorsoposterior edge simple; subapically with extended stridulatory patch. Metatibia ventrally with row of thin, erect setae. Abdominal ventrites 1–2 concave, densely punctate with coarse punctures and sparse plumose scales, interspaces microreticulate; ventrite 5 densely covered with erect to suberect plumose scales. Penis (Fig. 16b) with sides of body subparallel; apex subangular, medially extended into small tooth; with few long setae; apodemes 2.2× as long as body of penis; transfer apparatus spiniform, directed basad, held by anchor-shaped sclerites; ductus ejaculatorius without distinct bulbus. ***Intraspecific variation***. Length 1.80–1.98. Female rostrum slender, dorsally subglabrous, with submedian row of punctures and sublateral furrows. Female abdominal ventrites 1–2 almost flat, with sparse suberect setae; ventrite 5 flat with sparse plumose scales.

#### Material examined.

***Holotype*** (MZB, Cole.173.069): MZB0132 (GenBank OK481902), Indonesia, C-Sulawesi, Tojo Una-Una, Matako, Gn. Pompangeo, 01°35.235'S, 120°55.679'E to 01°35.258'S, 120°55.588'E, 1900 m, 27-II-2020, sifted. ***Paratypes*** (MZB, SMNK): Indonesia, C-Sulawesi, Tojo Una-Una, Matako, Gn. Pompangeo: 5 exx, MZB0131 (GenBank OK481903), same data as holotype; 1 ex, 01°35.235'S, 120°55.679'E to 01°35.258'S, 120°55.588'E, 1900 m, 27-II-2020, beaten; 2 exx, MZB0153 (GenBank OK481885), 01°35.197'S, 120°55.658'E to 01°35.127'S, 120°55.622'E, 2000 m, 01-III-2020, beaten; 1 ex, 01°35.235'S, 120°55.679'E to 01°35.258'S, 120°55.588'E, 1900 m, 26–27-II-2020, beaten; 1 ex, MZB0143 (GenBank OK481894), 01°35.359'S, 120°55.643'E to 01°35.581'S, 120°55.385'E, 1900 m, 29-II-2020, sifted.

#### Distribution.

C-Sulawesi Prov. (Mt Pompangeo). Elevation 1900–2000 m.

#### Biology.

In leaf litter of montane forest.

#### Etymology.

This epithet is a Latinized adjective based on Pomberimbe hill.

#### Notes.

*Trigonopteruspomberimbensis* sp. nov. was coded as “*Trigonopterus* sp. 1191”. This species belongs to the *T.barbipes*-group. It is closely related to *T.viduus* Riedel, which differs by weakly impressed elytral striae and 19.7–21.2% *cox1* p-distance.

### 17. Trigonopterus pompangeensis sp. nov.

Taxon classificationAnimaliaColeopteraCurculionidae

A71FD1BC-259A-56DD-A449-74FA11277EC0

http://zoobank.org/AD119D2E-9F79-4613-B54E-32B28582ADA4

#### Diagnostic description.

**Figure 17.**
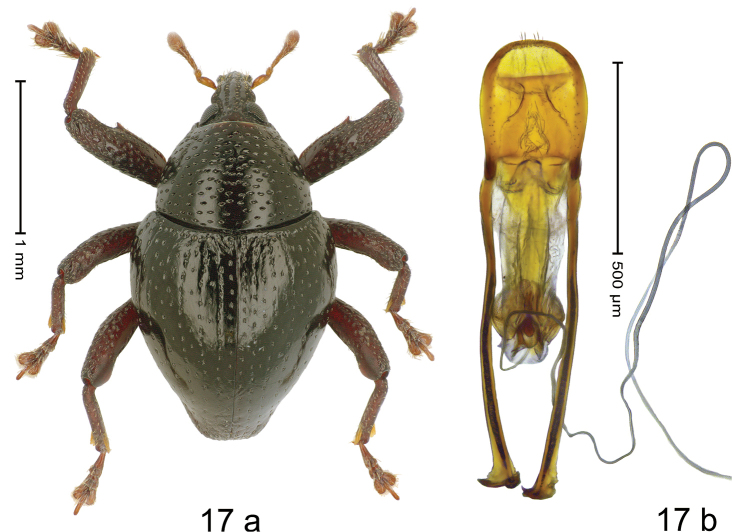
*Trigonopteruspompangeensis* sp. nov., holotype **a** habitus **b** penis.

***Holotype*. Male** (Fig. 17a). Length 2.16 mm. Color of antennae and legs ferruginous; remainder black. Body subovate; in dorsal aspect with weak constriction between pronotum and elytron; in profile dorsally convex. Rostrum dorsally with median costa and pair of somewhat irregular submedian ridges; epistome indistinct, subglabrous, apically with sparse suberect setae. Pronotum with ovate punctures of transverse orientation; interspaces subglabrous. Elytra with striae marked by rows of small punctures; sutural interval with additional row, other intervals subglabrous, with sparse minute punctures; basal margin with transverse row of denser punctures and wrinkles; stria 7 and 8 basally with somewhat coarser punctures. Femora dentate with small tooth; anterior surface densely punctate-rugose, microreticulate, each puncture with short recumbent seta. Meso- and metafemur with anteroventral ridge hardly crenate. Metafemur subapically with stridulatory patch. Abdominal ventrite 1–2 flat, subglabrous, with sparse punctures; ventrite 5 flat, densely punctate. Penis (Fig. 17b) with sides of body subparallel in basal half, converging in apical half, apex rounded, with few setae; apodemes 2.7× as long as body of penis; transfer apparatus spiniform, pointing apicad, held by pair of M-shaped sclerite; ductus ejaculatorius without bulbus. ***Intraspecific variation*.** Length 2.11–2.43. Female rostrum slender, dorsally subglabrous, with submedian row of punctures and sublateral furrows.

#### Material examined.

***Holotype*** (MZB, Cole.173.070): MZB0145 (GenBank OK481892), Indonesia, C-Sulawesi, Tojo Una-Una, Matako, Gn. Pompangeo, 01°35.603'S, 120°55.437'E to 01°35.406'S, 120°55.547'E, 1800 m, 29-II-2020, beaten. ***Paratypes*** (MZB, SMNK): Indonesia, C-Sulawesi, Tojo Una-Una, Matako, Gn. Pompangeo: 2 exx, MZB0239 (GenBank OK481814), MZB0240 (GenBank OK481813), same data as holotype; 1 ex, MZB0133 (GenBank OK481901), 01°35.235'S, 120°55.679'E to 01°35.258'S, 120°55.588'E, 1900 m, 27-II-2020, sifted; 3 exx, MZB0147 (GenBank OK481890), MZB0164 (GenBank OK481874), MZB0229 (GenBank OK481824), 01°35.197'S, 120°55.658'E to 01°35.154'S, 120°55.507'E, 1900 m, 28-II-2020, beaten; 1 ex, MZB0152 (GenBank OK481886), 01°35.359'S, 120°55.643'E to 01°35.581'S, 120°55.385'E, 1800 m, 29-II-2020, beaten; 1 ex, MZB0144 (GenBank OK481893), 01°35.235'S, 120°55.679'E to 01°35.258'S, 120°55.588'E, 1900 m, 26–27-II-2020, beaten; 4 exx, MZB0146 (GenBank OK481891), MZB0236 (GenBank OK481817), MZB0237 (GenBank OK481816), MZB0238 (GenBank OK481815), 01°35.197'S, 120°55.658'E to 01°35.127'S, 120°55.622'E, 2000 m, 01-III-2020, beaten.

#### Distribution.

C-Sulawesi Prov. (Mt Pompangeo). Elevation 1800–2000 m.

#### Biology.

On foliage in montane forest.

#### Etymology.

This epithet is a Latinized adjective based on Mt Pompangeo.

#### Notes.

*Trigonopteruspompangeensis* sp. nov. was coded as “*Trigonopterus* sp. 1198”. This species belongs to the *T.ovalipunctatus*-group. It is closely related to *T.ovalipunctatus* Riedel, but differs by a more densely punctate pronotum, the apically extended and converging penis, and 7.2–9.7% *cox1* p-distance.

### 18. Trigonopterus puspoi sp. nov.

Taxon classificationAnimaliaColeopteraCurculionidae

C7F9E218-5DE0-58AD-BC78-7AEF19981115

http://zoobank.org/9DFE5E8F-2010-4498-B534-FD4DD3265977

#### Diagnostic description.

**Figure 18.**
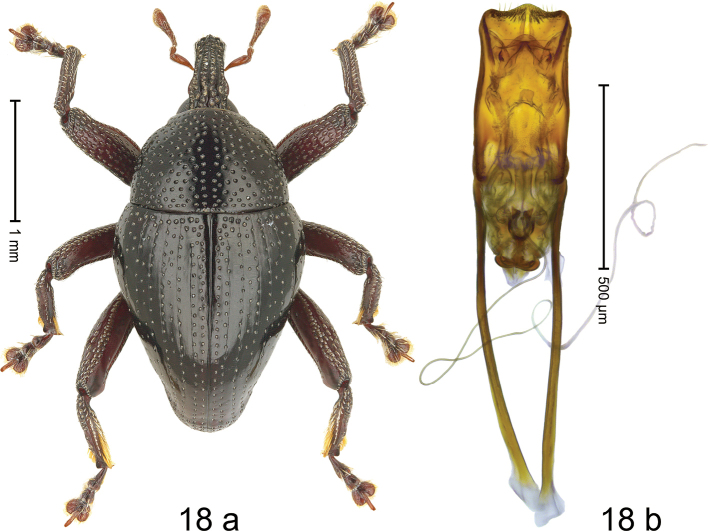
*Trigonopteruspuspoi* sp. nov., holotype **a** habitus **b** penis.

***Holotype*** (MZB). Male (Fig. 18a). Length 2.75 mm. Color of antennae ferruginous; legs dark ferruginous; remainder black. Body subovate; in dorsal aspect with weak constriction between pronotum and elytron; in profile dorsally convex. Rostrum with median and pair of submedian ridges; epistome distinct, posteriorly with subangulate ridge bearing five denticles. Pronotum with very weak subapical constriction; disk densely punctate; median line impunctate; interspaces between punctures subglabrous, subequal to punctures’ diameter. Elytra with striae marked by rows of small punctures and fine hairlines; along basal margin with transverse row of denser punctures; stria 8 along humerus with seven coarse punctures; intervals subglabrous with interspersed punctures. Femora edentate, with distinct anteroventral ridge weakly crenate. Metafemur with dorsoposterior edge crenate; subapically with stridulatory patch. Posterior surface of metatibia with in apical half covered with long subrecumbent setae. Abdominal ventrites 1–2 concave, microreticulate, with sparse punctures; behind metacoxa with angular knob; ventrite 5 concave, subglabrous, sublaterally and subapically with sparse erect scales. Penis (Fig. 18b) with sides of body subparallel; apex symmetrical, with median triangular extension and sparse setae; apodemes 2.0× as long as body of penis; transfer apparatus Ʊ-shaped; ductus ejaculatorius without bulbus. ***Intraspecific variation*.** Length 2.48–3.36 mm. Female rostrum smooth and flat, epistome indistinct. Female metatibia subbasally dorsally somewhat widened, denticulate. Female abdominal ventrite 1 and 2 concave, subglabrous, laterally with few setae. Female abdominal ventrite 5 flat, medially glabrous, sublaterally and subapically punctate.

#### Material examined.

***Holotype*** (MZB, Cole.173.071): MZB0052 (GenBank OK481963), Indonesia, C-Sulawesi Prov, Toli-Toli, Gn. Dako, 01°03.967'N, 120°53.692'E to 01°03.782'N, 120°53.934'E, 840–970 m, 05-VII-2018, beaten. ***Paratypes*** (MZB, SMNK): Indonesia, Sulawesi Tengah, Toli-Toli, Gn. Dako: 1 ex, MZB0285 (GenBank OK481771), 01°03.967'N, 120°53.692'E to 01°03.782'N, 120°53.934'E, 840–970 m, 03-VII-2018, beaten; 1 ex, MZB0272 (GenBank OK481784), 01°04.1812'N, 120°53.5652'E to 01°3.9665'N, 120°53.6915'E, 720–830 m, 01-VII-2018, beaten; 2 exx, MZB0085 (GenBank OK481932), 01°03.574'N, 120°54.032'E to 01°03.323'N, 120°54.195'E, 1100–1200 m, 06-VII-2018, beaten; 1 ex, MZB0093 (GenBank OK481924), 01°03.574N, 120°54.032'E to 01°03.782N, 120°53.934'E, 830–1000 m, 05-VII-2018, beaten; 6 exx, MZB0260 (GenBank OK481793), MZB0261 (GenBank OK481792), MZB0262 (GenBank OK481791), MZB0263 (GenBank OK481790), MZB0287 (PCR failed), MZB0288 (GenBank OK481770), 01°03.782N, 120°53.934'E to 01°03.574N, 120°54.032'E, 970–1140 m, 06-VII-2018, beaten; 2 exx, MZB0259 (GenBank OK481794), MZB0284 (GenBank OK481772, 01°03.782'N, 120°53.934'E to 01°03.574'N, 120°54.032'E, 970–1100 m, 06-VII-2018, beaten; 7 exx, MZB0264 (GenBank OK481789), 01°03.574'N, 120°54.032'E to 01°03.157'N, 120°54.195'E, 1100–1120 m, 06-VII-2018, beaten; 1 ex, MZB0286 (PCR failed), 01°03.782'N, 120°53.934'E to 01°02.977'N, 120°55.001'E, 1250–1750 m, 11-VII-2018.

#### Distribution.

C-Sulawesi Prov. (Mt Dako). Elevation 830–1250 m.

#### Biology.

On foliage in montane forest.

#### Etymology.

This species is named in honor of Saleh Poespo, grandfather of the first author, and for his pioneering animal husbandry science in Indonesia. An invariable genitive.

#### Notes.

*Trigonopteruspuspoi* sp. nov. was coded as “*Trigonopterus* sp. 1113” (Narakusumo et al. 2020) and belongs to the *T.palopensis*-group. It is closely related to *T.tolitoliensis* sp. nov., from which it differs by its simple penis surface, the pubescence of the metatibia, and a 16.4–18.1% p-distance of its *cox1* sequence.

### 19. Trigonopterus rosichoni sp. nov.

Taxon classificationAnimaliaColeopteraCurculionidae

56159AA7-C44C-5BA7-A0C6-B1254F34F792

http://zoobank.org/B32661A6-A47A-40DE-8B3E-DBCE02563F99

#### Diagnostic description.

**Figure 19.**
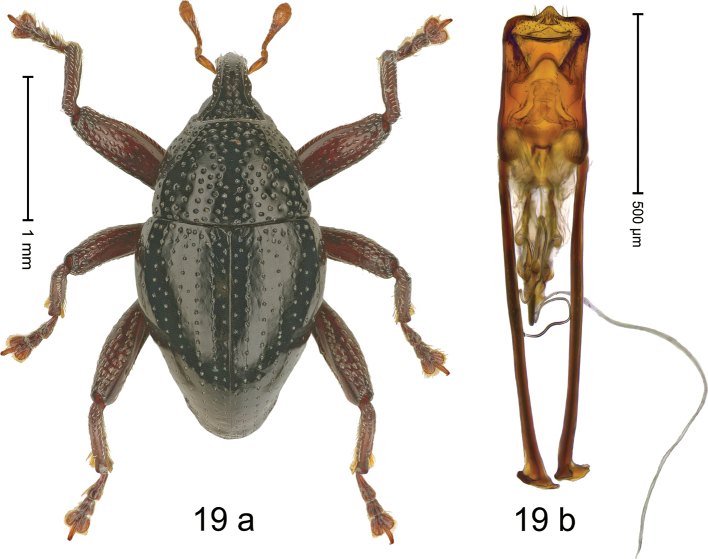
*Trigonopterusrosichoni* sp. nov., holotype **a** habitus **b** penis.

***Holotype*. Male** (Fig. 19a). Length 2.40 mm. Color of antennae and legs ferruginous; remainder black. Body subovate; in dorsal aspect with weak constriction between pronotum and elytron; in profile dorsally convex. Rostrum dorsally with broad median costa and pair of submedian ridges; intervening furrows with sparse rows of subrecumbent setae; apical 1/3 subglabrous, with few punctures and with sparse suberect setae. Pronotum with disk densely punctate with coarse punctures; median line impunctate; interspaces between punctures subglabrous; laterally in basal 1/3 impunctate. Elytra with striae marked by rows of punctures and very fine hairlines; basal margin bordered by transverse row of punctures; intervals flat, with few interspersed punctures. Femora edentate; anteroventral ridges simple. Metafemur with dorsoposterior edge weakly crenate; subapically with stridulatory patch. Abdominal ventrites 1–2 concave, subglabrous; behind metacoxa with angular knob; ventrite 5 with deep subglabrous pit, subparallel lateral ridges and apical margin microreticulate, punctulate. Penis (Fig. 19b) with sides of body subparallel; apex with median angulate extension, with sparse setae; apodemes 2.6× as long as body of penis; transfer apparatus flagelliform; ductus ejaculatorius without bulbus. ***Intraspecific variation*.** Length 2.28–2.80 mm. Female rostrum dorsally smooth and flat. Female abdominal ventrite 5 flat, subglabrous, with sparse minute punctures.

#### Material examined.

***Holotype*** (MZB, Cole.173.072): MZB0094 (GenBank OK481923), Indonesia, C-Sulawesi, Toli-Toli, Gn. Dako, 01°03.782N, 120°53.934'E to 01°02.977N, 120°55.001'E, 1250–1750 m, 11-VII-2018, beaten. ***Paratypes*** (MZB, SMNK): Indonesia, C-Sulawesi, Toli-Toli, Gn. Dako: 6 exx, MZB0193 (GenBank OK481857), MZB0194 (GenBank OK481856), MZB0249 (GenBank OK481804), same data as holotype; 3 exx, MZB0092 (GenBank OK481925), MZB0056 (GenBank OK481959), MZB0252 (GenBank OK481801), 01°03.181N, 120°54.607'E to 01°02.977N, 120°55.001'E, 1400–1750 m, 07-VII-2018, beaten; 2 exx, MZB0253 (GenBank OK481800), MZB0255 (GenBank OK481798), 01°02.977'N, 120°55.010'E to 01°03.782N, 120°53.934'E, 970–1750 m, 11-VII-2018, beaten.

#### Distribution.

C-Sulawesi Prov. (Mt Dako). Elevation ca. 1400–1750 m.

#### Biology.

On foliage in montane forest.

#### Etymology.

This species is named in honor of Rosichon Ubaidillah, curator and researcher of Hymenoptera at MZB. An invariable genitive.

#### Notes.

*Trigonopterusrosichoni* sp. nov. was coded as “*Trigonopterus* sp. 1193”. It belongs to the *T.satyrus*-group and is closely related to *T.ancora* sp. nov. from which it differs by the shape of the transfer apparatus and 9.4–9.6% p-distance of its *cox1* sequence.

### 20. Trigonopterus rubidus sp. nov.

Taxon classificationAnimaliaColeopteraCurculionidae

C2E04A82-6F08-5D43-9E65-DD1EF7BC06C5

http://zoobank.org/7464F2DE-28B8-4563-9EAF-C0501EFB4C93

#### Diagnostic description.

**Figure 20.**
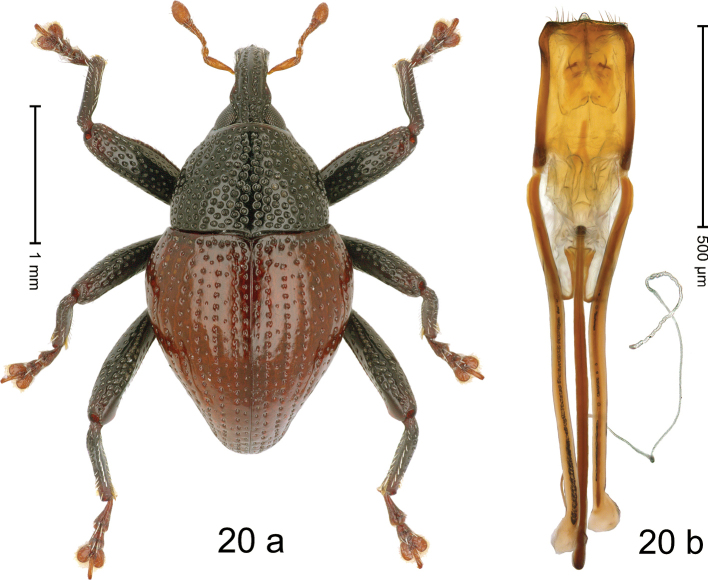
*Trigonopterusrubidus* sp. nov., holotype **a** habitus **b** penis.

***Holotype*.Male** (Fig. 20a). Length 2.50 mm. Color of antennae and elytra ferruginous; remainder black. Body subovate; in dorsal aspect with distinct constriction between pronotum and elytron, in profile dorsally convex. Rostrum dorsally with median and pair of submedian costae; intervening furrows with rows of coarse punctures and small setae; epistome indistinct, subglabrous with suberect setae. Pronotum with disk densely punctate; interspaces between large punctures subglabrous, subequal or smaller than punctures´ diameter. Elytra with striae distinct, marked by rows of punctures; intervals subglabrous, with few scattered punctures; basal margin bordered by row of coarse, dense punctures. Meso- and metafemur with anteroventral ridge weakly crenate. Metafemur subapically with stridulatory patch. Meso- and metatibia subglabrous, with sparse erect setae. Abdominal ventrites 1–2 concave, subglabrous, with coarse punctures; ventrite 5 with shallow impression, densely punctate. Penis (Fig. 20b) with sides of body subparallel; apex broadly angulate, with sparse setae; apodemes 3.0× as long as body of penis; transfer apparatus flagelliform, curved, pointing basad, basally supported by pair of L-shaped sclerites; ductus ejaculatorius basally markedly sclerotized and somewhat swollen, curving around apodeme tips, then becoming thin and membranous. *Intraspecific variation*. Length 2.16–2.93 mm; elytral coloration orange to dark ferruginous. Female rostrum more slender, dorsally subglabrous, with rows of punctures. Female abdominal ventrites 1–2 weakly concave, punctate. Female abdominal ventrite 5 flat, densely punctate.

#### Material examined.

***Holotype*** (MZB, Cole.173.073): MZB0161 (GenBank OK481877), Indonesia, C-Sulawesi, Toli-Toli, Gn. Dako, 01°03.389'N, 120°55.524'E to 01°03.567'N, 120°56.032'E, 1900–2200 m, 10-VII-2018, beaten. ***Paratypes*** (MZB, SMNK): Indonesia, C-Sulawesi, Toli-Toli, Gn. Dako: 40 exx, MZB0216 (GenBank OK481837), same as holotype; 5 exx, MZB0066 (GenBank OK481951), 01°03.782'N, 120°53.934'E to 01°02.977'N, 120°55.001'E, 1250–1750 m, 11-VII-2018, beaten; 18 exx, 01°02.977'N, 120°55.967'E to 01°03.210'N, 120°55.297'E, 1700–1800 m, 08–10-VII-2018, beaten; 6 exx, MZB0067 (GenBank OK481950), MZB0068 (GenBank OK481949), 01°03.174'N, 120°55.272'E to 01°03.389'N, 120°55.524'E, 1800–1900 m, 10-VII-2018, beaten; 31 exx, MZB0069 (GenBank OK481948), 01°03.174'N, 120°55.272'E to 01°03.389'N, 120°55.524'E, 1800–1900 m, 10-VII-2018, beaten; 5 exx, 01°03.174'N, 120°55.272'E to 01°03.389'N, 120°55.524'E, 1800–1900 m, 08–10-VII-2018, beaten.

#### Distribution.

C-Sulawesi Prov. (Mt Dako). Elevation 1700–1900 m.

#### Biology.

On foliage in montane forest.

#### Etymology.

This epithet is the Latin adjective *rubidus*, -*a*, -*um* (reddish) referring to the elytral color.

#### Notes.

*Trigonopterusrubidus* sp. nov. was coded as “*Trigonopterus* sp. 1199”. This species belongs to the *T.tatorensis*-group. It is closely related to *T.tatorensis* Riedel, from which it differs by denser pronotal punctures, its reddish elytral color and 9.5–9.9% *cox1* p-distance.

### 21. Trigonopterus sarinoi sp. nov.

Taxon classificationAnimaliaColeopteraCurculionidae

D13646E3-0AD1-5865-816F-599FC4DCE658

http://zoobank.org/863640D7-FD62-4CF6-9A97-264A73A7C09F

#### Diagnostic description.

**Figure 21.**
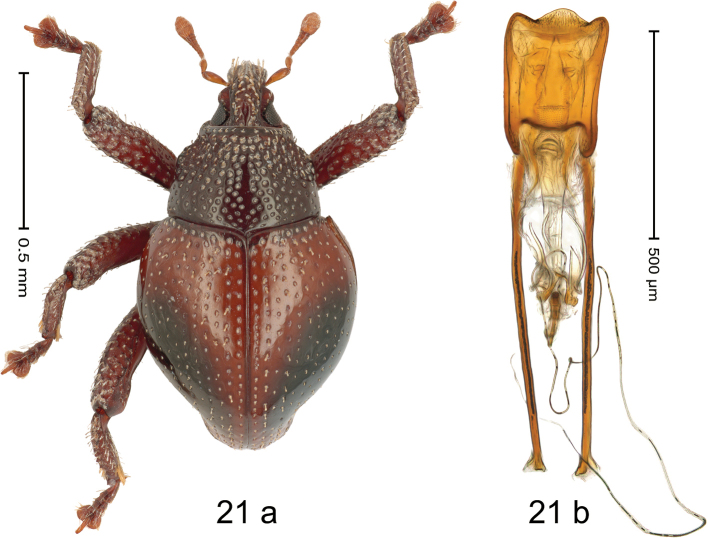
*Trigonopterussarinoi* sp. nov., holotype **a** habitus **b** penis.

***Holotype*. Male** (Fig. 21a). Length 2.40 mm. Color of antennae and elytral base ferruginous; legs dark ferruginous; head, thorax and elytral sides black. Body subovate; in dorsal aspect with distinct constriction between pronotum and elytron; in profile dorsally convex. Rostrum at base dorsally swollen, markedly bent ventrad; with lateral flanges in front of eyes; dorsally with distinct median carina and pair of submedian ridges; intervening furrows each with row of erect, clavate scales; epistome indistinct. Pronotum with distinct subapical constriction; disk densely punctate; interspaces subglabrous; with subglabrous median costa; in apical half with erect, clavate scales. Elytra irregularly punctate; striae indistinct; interspaces subglabrous; some punctures with suberect, piliform to subclavate scale, in some areas missing or abraded. Meso- and metafemur with anteroventral ridge crenate, ending with small tooth; anterior surface coarsely punctate-reticulate, with erect subclavate scales. Metafemur with dorsoposterior edge serrate; subapically with stridulatory patch. Metatibia with dorsal edge serrate. Abdominal ventrites 1–2 concave, subglabrous, with few scattered erect scales; ventrite 5 almost flat, with broad shallow impression, microreticulate, with sparse suberect scales. Penis (Fig. 21b) with sides of body diverging; apex medially with subangular extension, without setae; apodemes 2.0× as long as body of penis; transfer apparatus flagelliform, forming a full coil, basally held by lyriform sclerite; ductus ejaculatorius with indistinct bulbus.

#### Material examined.

***Holotype*** (MZB, Cole.173.074): MZB0106 (GenBank OK481913), Indonesia, C-Sulawesi, Sigi, Kolagi, Namu, Gn. Torompupu, 01°24.637'S, 119°57.597'E, 800 m, 15–27-XI-2017, sifted. Right thorax and elytron cracked, right mid- and hind leg glued separately to card.

#### Distribution.

C-Sulawesi Prov. (Gn. Torompupu). Elevation ca. 800 m.

#### Biology.

In leaf litter.

#### Etymology.

This species is named in honor of Sarino, technician working at the Coleoptera collection of LIPI-MZB. An invariable genitive.

#### Notes.

*Trigonopterussarinoi* sp. nov. was coded as “*Trigonopterus* sp. 1208”. This species belongs to the *T.lampros*-group. It is closely related to *T.yoda* Riedel, which differs by its black-bronze elytral color and a 19.3% *cox1* p-distance.

### 22. Trigonopterus sutrisnoi sp. nov.

Taxon classificationAnimaliaColeopteraCurculionidae

8A2362EA-A9C6-535B-B581-4A9EAC1B612C

http://zoobank.org/963BE7BE-53F4-4473-9485-CCE33EC99E53

#### Diagnostic description.

**Figure 22.**
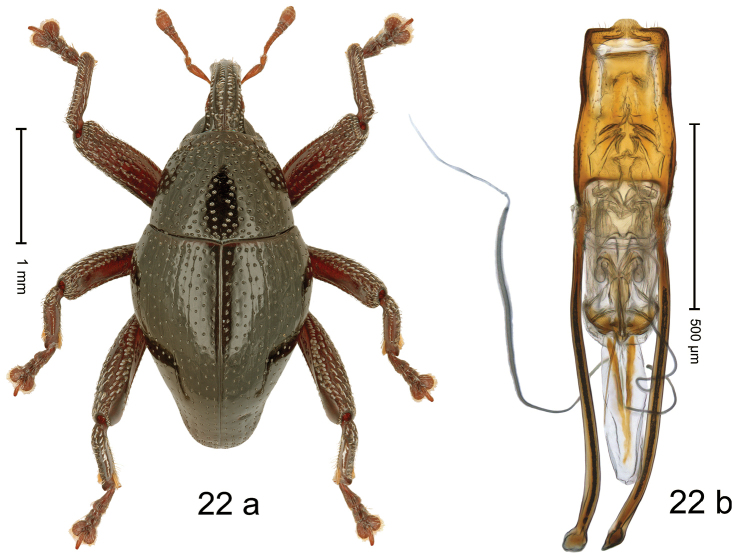
*Trigonopterussutrisnoi* sp. nov., holotype **a** habitus **b** penis.

***Holotype*. Male** (Fig. 22a). Length 2.88 mm. Color of antennae ferruginous, legs dark ferruginous, remainder black. Body subrhomboid; in profile dorsally convex. Rostrum with median and pair of submedian carinae; intervening furrows with rows of suberect scales; epistome indistinct, sparsely setose; profile in basal 1/3 dorsally swollen, markedly convex to forehead. Pronotum with disk densely punctate, interspace subglabrous; median line impunctate. Elytra irregularly punctate with small punctures; interspaces subglabrous; striae 2–5 marked by fine hairlines. Femora edentate; with anteroventral ridge crenate. Metafemur dorsally with suberect silvery scales, dorsoposterior edge denticulate; subapically with stridulatory patch. Metatibia with dorsal edge very weakly denticulate, subapically with constriction; ventrally with sparse row of long setae. Meso- and metathorax ventrally densely squamose with erect plumose scales. Abdominal ventrites 1–2 concave, subglabrous, almost impunctate, sublaterally with sparse suberect scales; ventrite 5 flat, densely punctate, punctures with dense erect scales, interspaces microreticulate. Penis (Fig. 22b) with sides of body subparallel, near middle with very shallow constriction; apex with median extension, with sparse setae; apodemes 2.0× as long as body of penis; transfer apparatus long spiniform, pointing basad, with complex supporting sclerites; basal sclerite elongate V-shaped; ductus ejaculatorius with bulbus. ***Intraspecific variation*.** Length 2.48–2.88 mm. Female rostrum slender, in apical half dorsally subglabrous, with submedian row of punctures and sublateral furrows. Female abdominal ventrite 5 flat, sparsely punctate, with sparse scales.

#### Material examined.

***Holotype*** (MZB, Cole.173.075): MZB0203 (GenBank OK481848), Indonesia, C-Sulawesi, Toli-Toli, Gn. Dako, 01°04.181'N, 120°53.565'E to 01°03.967'N, 120°53.692'E, 835–970 m, 03-VII-2018, beaten. ***Paratype*** (SMNK): MZB0097 (GenBank OK481920), Indonesia, C-Sulawesi, Toli-Toli, Gn. Dako, 01°04.1812'N, 120°53.5652'E to 01°3.9665'N, 120°53.6915'E, 720–830 m, 01-VII-2018, beaten.

#### Distribution.

C-Sulawesi Prov. (Mt Dako). Elevation ca. 720–970 m.

#### Biology.

On foliage in lower montane forest.

#### Etymology.

This epithet is named in honor of Hari Sutrisno, curator of moths and researcher at MZB. An invariable genitive.

#### Notes.

*Trigonopterussutrisnoi* sp. nov. was coded as “*Trigonopterus* sp. 1206”. This species belongs to the *T.toraja*-group. It is related to *T.toboliensis* sp. nov., which differs by its lateral extensions of the penis and a *cox1* p-distance of 15.3–15.5%.

### 23. Trigonopterus tanah sp. nov.

Taxon classificationAnimaliaColeopteraCurculionidae

E4137F41-DBBC-50E4-895B-95DEFEBE6B3B

http://zoobank.org/E32B51C0-0A96-49D3-9442-3D0513B57B40

#### Diagnostic description.

**Figure 23.**
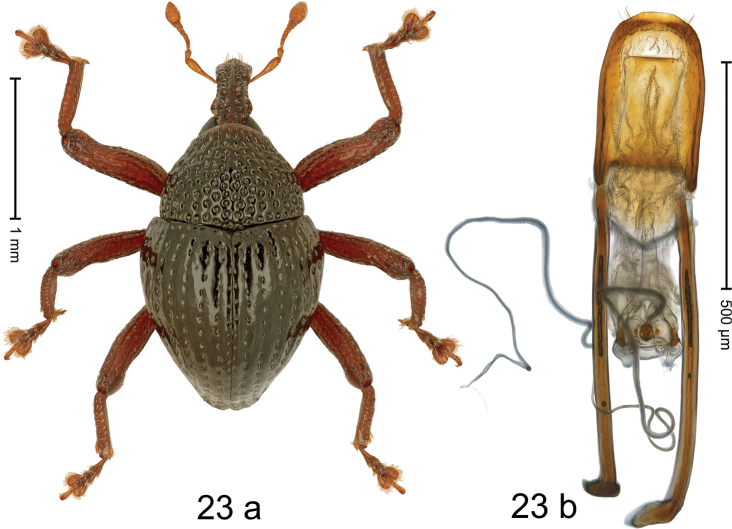
*Trigonopterustanah* sp. nov., holotype **a** habitus **b** penis.

***Holotype*. Male** (Fig. 23a). Length 2.13 mm. Color of antennae yellowish; legs ferruginous; remainder black. Body subovate; in dorsal aspect with weak constriction, in profile with marked constriction between pronotum and elytron. Rostrum dorsally with median costa and pair of submedian ridges; intervening furrows filled with rows of coarse punctures containing each one indistinct seta; epistome simple, subglabrous. Pronotum with disk dorsally swollen, densely coarsely punctate-reticulate; interspaces between punctures subglabrous. Elytra with striae impressed, with dense rows of deep punctures; sutural interval with row of minute punctures, other intervals subglabrous, costate; basal margin bordered by transverse row of punctures. Profemur with anteroventral ridge simple; meso- and metafemur with denticle in apical 1/2; anterior surface of femora with longitudinal wrinkles, weakly punctate. Metafemur subapically with stridulatory patch. Abdominal ventrites 1–2 microreticulate, concave, with coarse punctures; ventrite 5 almost flat, microreticulate, sparsely punctate. Penis (Fig. 23b) with sides of body subparallel; apex rounded, sublaterally with sparse setae; apodemes 2.3 X as long as body of penis; transfer apparatus dentiform, directed basad in repose, flanked by pair of small sclerites; ductus ejaculatorius with indistinct bulbus.

#### Material examined.

***Holotype*** (MZB, Cole.173.056): MZB0202 (GenBank OK481849), Indonesia, C-Sulawesi, Toli-Toli, Gn. Dako, 01°03.512'N, 120°54.054'E, 1100–1200 m, 13-VII-2018, sifted.

#### Distribution.

C-Sulawesi Prov. (Mt Dako). Elevation ca. 1100–1200 m.

#### Biology.

In leaf litter of montane forest.

#### Etymology.

This epithet is the Indonesian word for “soil” and a noun in apposition. It refers to the species´ lifestyle on the ground among leaf litter.

#### Notes.

*Trigonopterustanah* sp. nov. was coded as “*Trigonopterus* sp. 1234”. It is closely related to *T.darwini* Riedel, from which it can be distinguished by its coarser sculpture, and the subparallel body of the penis. The *cox1* p-distance of both species is 10.8%.

### 24. Trigonopterus tejokusumoi sp. nov.

Taxon classificationAnimaliaColeopteraCurculionidae

6B7B344D-2D27-59F5-A105-B899043474B7

http://zoobank.org/7AD6A5C2-873A-467B-98CD-220501419A60

#### Diagnostic description.

**Figure 24.**
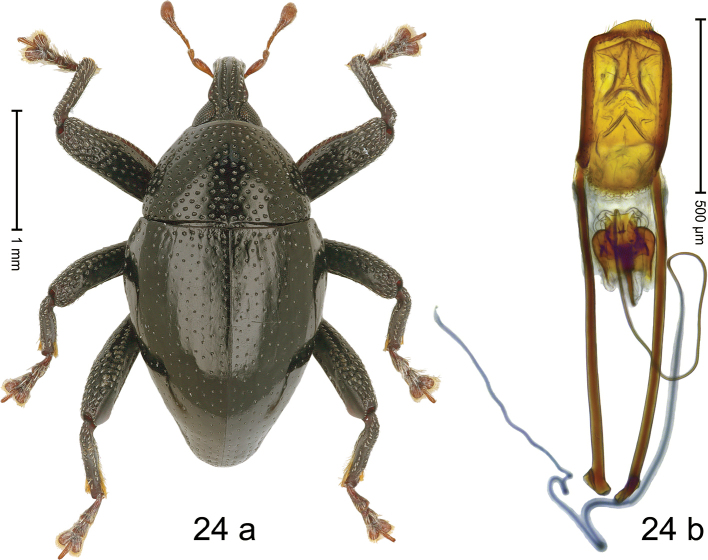
*Trigonopterustejokusumoi* sp. nov., holotype **a** habitus **b** penis.

***Holotype*. Male** (Fig. 24a). Length 2.90 mm. Color of antennae and tarsi ferruginous, remainder black. Body subovate; in dorsal aspect with weak constriction between pronotum and elytron; in profile dorsally convex. Rostrum dorsally with median costa and pair of submedian ridges; intervening furrows with sparse rows of suberect scales; epistome indistinct, subglabrous. Eyes medially approximate. Pronotum with disk densely punctate, laterally punctures larger; interspaces subglabrous. Elytra densely irregularly punctate with small punctures; striae indistinct; hardly visible; interspaces subglabrous; stria 8 along humerus with row of six coarse punctures. Femora edentate; anterior and dorsal surface coarsely punctate, reticulate, each puncture containing silvery elongate scale. Meso- and metafemur with anteroventral ridge crenate; metafemur subapically with stridulatory patch. Metatibia in apical half ventrally with fringe of long setae. Abdominal ventrites 1–2 concave, dull, with sparse punctures, each bearing suberect scale; ventrite 5 with shallow impression, microreticulate, dull, with sparse suberect scales. Penis (Fig. 24b) with sides of body subparallel; apex asymmetrical, obtuse median extension shifted to the left, with sparse setae; apodemes 2.1× as long as body of penis; transfer apparatus spiniform, supported by plate-like sclerite; ductus ejaculatorius with indistinct bulbus. ***Intraspecific variation***. Length 2.38–3.00 mm. Female body more slender. Female rostrum slender, dorsally subglabrous, with submedian row of punctures and sublateral furrows. Female abdominal ventrite 5 flat, punctate, with suberect scales.

#### Material examined.

***Holotype*** (MZB, Cole.173.076): MZB0137 (GenBank OK481897), Indonesia, C-Sulawesi, Tojo Una-Una, Matako, Gn. Pompangeo, 01°35.215'S, 120°55.560'E to 01°35.079'S, 120°55.49'E, 1900 m, 28-II-2020, beaten. ***Paratypes*** (MZB, SMNK): Indonesia, C-Sulawesi, Tojo Una-Una, Matako, Gn. Pompangeo 7 exx, same data as holotype; 13 exx, MZB0139 (GenBank OK481895), MZB0206 (GenBank OK481847), 01°35.603'S, 120°55.437'E to 01°35.406'S, 120°55.547'E, 1800 m, 29-II-2020, beaten; 15 exx, MZB0266 (PCR failed), MZB0267 (PCR failed), MZB0268 (PCR failed), 01°35.359'S, 120°55.643'E to 01°35.581'S, 120°55.385'E, 1800 m, 29-II-2020, beaten; 8 exx, 01°35.264'S, 120°55.588'E to 01°35.339'S, 120°55.599'E, 1900 m, 27-II-2020, beaten; 12 exx, 01°35.264'S, 120°55.588'E to 01°35.339'S, 120°55.599'E, 1900 m, 26–27-II-2020, beaten; 4 exx, MZB0211 (GenBank OK481842), MZB0212 (GenBank OK481841), 01°35.074'S, 120°55.467'E to 01°35.154'S, 120°55.507'E, 1900 m, 28-II-2020, beaten; 26 exx, MZB0138 (GenBank OK481896), MZB0265 (GenBank OK481788), 01°35.197'S, 120°55.658'E to 01°35.127'S, 120°55.622'E, 2000 m, 01-III-2020, beaten; 1 ex, 01°35.197'S, 120°55.658'E to 01°35.127'S, 120°55.622'E, 2000 m, 01-III-2020, sifted.

#### Distribution.

C-Sulawesi Prov. (Mt Pompangeo). Elevation 1800–2000 m.

#### Biology.

On foliage in montane forest.

#### Etymology.

This species is named in honor of Slamet Tedjokoesoemo, pioneer of veterinary science in Indonesia and grandfather of the first author. An invariable genitive.

#### Notes.

*Trigonopterustejokusumoi* sp. nov. was coded as “*Trigonopterus* sp. 1200”. This species belongs to the *T.barbipes*-group. It is most closely related to *T.barbipes* Riedel, but differs by smaller and more irregular elytral punctures, a peculiar obtuse apex of the penis and a 16% *cox1* p-distance.

### 25. Trigonopterus toboliensis sp. nov.

Taxon classificationAnimaliaColeopteraCurculionidae

A7994E65-AE69-5D8A-8DA6-8B4605DF2030

http://zoobank.org/3CD000BF-2C7C-4FA4-85B0-3174D02272D4

#### Diagnostic description.

**Figure 25.**
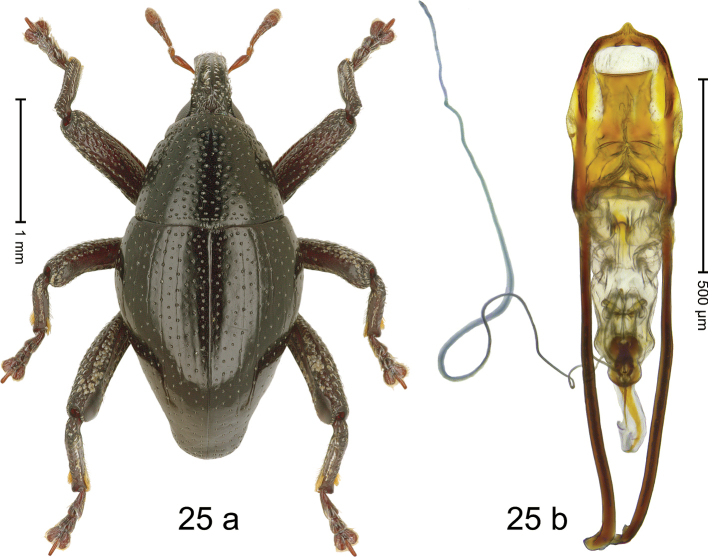
*Trigonopterustoboliensis* sp. nov., holotype **a** habitus **b** penis.

***Holotype*. Male** (Fig. 25a). Length 2.90 mm. Color of antennae ferruginous, legs dark ferruginous, remainder black. Body subrhomboid; in profile dorsally convex. Rostrum with median and pair of submedian carinae; intervening furrows with rows of suberect scales; epistome indistinct, sparsely setose; profile in basal 1/3 dorsally swollen, markedly convex to forehead. Pronotum with disk densely punctate, interspace subglabrous; median line impunctate. Elytra irregularly punctate with small punctures; interspaces subglabrous; striae 2–6 marked by fine hairlines. Femora edentate; with anteroventral ridge crenulate. Metafemur dorsally with suberect silvery scales, dorsoposterior edge denticulate; subapically with stridulatory patch. Metatibia with dorsal edge very weakly denticulate, subapically with constriction. Abdominal ventrites 1–2 concave, subglabrous, almost impunctate, with sparse suberect scales; ventrite 5 flat, densely punctate, punctures with suberect scales, interspaces microreticulate. Penis (Fig. 25b) with sides of body subparallel, near middle body with lateral flanges; apex with median triangular extension, without setae; apodemes 2.2× as long as body of penis; transfer apparatus spiniform; ductus ejaculatorius with indistinct bulbus. ***Intraspecific variation*.** Length 2.90–3.09 mm. Female rostrum slender, in apical half dorsally subglabrous, with submedian row of punctures and sublateral furrows. Female ventrite 5 flat, sparsely punctate, subapically with sparse scales.

#### Material examined.

***Holotype*** (MZB, Cole.173.077): MZB0126 (GenBank OK481908), Indonesia, C-Sulawesi, Palu, Donggala – Toboli, Kebun Kopi, 00°43.256'S, 119°56.759'E, 850 m, 03-III-2020, beaten. ***Paratypes*** (MZB, SMNK): MZB0127 (GenBank OK481907), MZB0213 (GenBank OK481840), MZB0214 (GenBank OK481839), MZB0215 (GenBank OK481838), same data as holotype; 4 exx, ARC7157 (GenBank OK481769), Palu, Palolo, Kamarora, trail to waterfall, ca. 01°12.541'S, 120°09.648'E, 700 m, 23–27-VIII-1997, beaten.

#### Distribution.

C-Sulawesi Prov. (Palu, Palolo). Elevation 700–850 m.

#### Biology.

On foliage in montane forest.

#### Etymology.

This epithet is a Latinized adjective based on Toboli village.

#### Notes.

*Trigonopterustoboliensis* sp. nov. was coded as “*Trigonopterus* sp. 1192”. This species belongs to the *T.toraja*-group. It is closely related to *T.ampanensis* Riedel from which it differs by the shape and position of the lateral extensions of the penis and a *cox1* p-distance of 14.5–14.7%.

### 26. Trigonopterus tolitoliensis sp. nov.

Taxon classificationAnimaliaColeopteraCurculionidae

A126C2FF-E411-55BA-9283-BD7C3FC71DB8

http://zoobank.org/0E1883D2-0FCF-446C-BC9A-8B0BB603DDDF

#### Diagnostic description.

**Figure 26.**
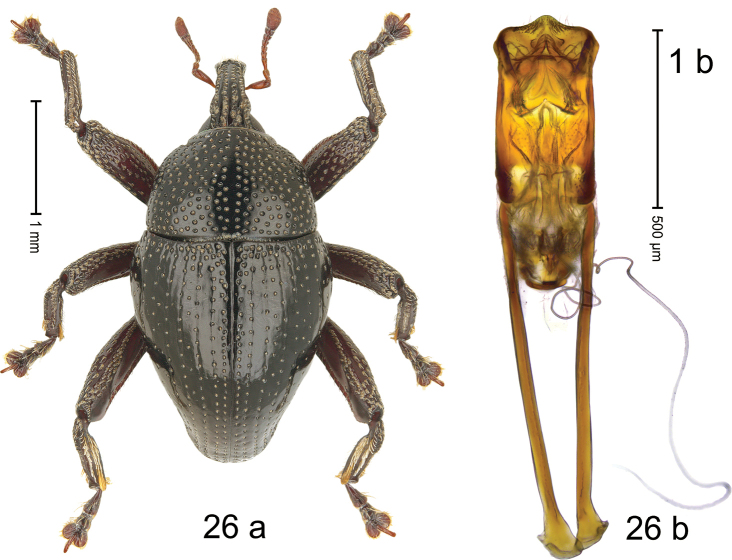
*Trigonopterustolitoliensis* sp. nov., holotype **a** habitus **b** penis.

***Holotype*. Male** (Fig. 26a). Length 3.00 mm. Color of antennae ferruginous; legs dark ferruginous; remainder black. Body subovate; in dorsal aspect with weak constriction between pronotum and elytron; in profile dorsally convex. Rostrum dorsally with median and pair of submedian costae separated by row of coarse punctures; epistome with surface subglabrous and suberect setae, posteriorly with three denticles. Pronotum with weak subapical constriction; disk densely punctate; median line impunctate; interspaces between punctures subglabrous, subequal to punctures’ diameter. Elytra subglabrous, with striae marked by rows of small punctures and fine hairlines; along basal margin with transverse row of denser punctures; stria 8 along humerus with six coarse punctures; intervals subglabrous. Femora edentate, with distinct anteroventral ridge. Metafemur with dorsoposterior edge crenate; subapically with stridulatory patch. Posterior surface of metatibia with rows of long suberect spatulate scales. Abdominal ventrites 1–2 concave, dull-shagreened, with sparse punctures; behind metacoxa with angular knob; ventrite 5 concave, subglabrous, sublaterally with sparse erect scales. Penis (Fig. 26b) with sides of body subparallel; apex symmetrical, with median subangular extension and sparse setae; dorsally body in front of middle with pair of brushes of long setae; apodemes 1.9× as long as body of penis; transfer apparatus Y-shaped; ductus ejaculatorius without bulbus. ***Intraspecific variation***. Length 2.08–3.16 mm. Female rostrum in apical half slender, dorsally subglabrous, with submedian rows of punctures and sublateral furrows; female epistome indistinct. Female abdominal ventrites 1–2 weakly concave, with sparse minute punctures; female ventrite 5 weakly concave, with scattered punctures and setae.

#### Material examined.

***Holotype*** (MZB, Cole.173.078): MZB0050 (GenBank OK481965), Indonesia, C-Sulawesi Prov., Toli-Toli, Gn. Dako, Base camp 1, 01°03.782'N, 120°53.934'E to 01°03.574'N, 120°54.032'E, 970–1100 m, 05–06-VII-2018, beaten. ***Paratypes*** (MZB, SMNK): Indonesia, C-Sulawesi Prov., Toli-Toli, Gn. Dako: 52 exx, same as holotype; 1 ex, 01°04.181'N, 120°53.565'E to 01°03.967'N, 120°53.692'E, 720–830 m, 01-VII-2018, beaten; 1 ex, 01°03.967'N, 120°53.692'E to 01°03.782'N, 120°53.934'E, 830–970 m, 01-VII-2018, beaten; 1 ex, 01°03.967'N, 120°53.692'E to 01°03.782'N, 120°53.934'E, 830–970 m, 05-VII-2018, beaten; 3 exx, 01°03.967'N, 120°53.692'E to 01°03.782'N, 120°53.934'E, 830–970 m, 03-VII-2018, beaten; 1 ex, MZB0271 (GenBank OK481785), 01°03.782'N, 120°53.934'E, 970 m, 04-VII-2018, beaten; 23 exx, MZB0095 (GenBank OK481922), MZB0098 (GenBank OK481919), MZB0096 (GenBank OK481921), MZB0273 (GenBank OK481783), MZB0274 (GenBank OK481782), MZB0275 (GenBank OK481781), 01°03.697'N, 120°53.991'E, 970 m, 07-VII-2018, beaten; 12 exx, MZB0086 (GenBank OK481931) 01°03.574'N, 120°54.032'E to 01°03.782'N, 120°53.934'E, 830–1000 m, 05-VII-2018, beaten; 7 exx, 01°03.782'N, 120°53.934'E to 01°03.967N, 120°53.692'E, 970–1000 m, 05-VII-2018, beaten; 66 exx, MZB0087 (GenBank OK481930), MZB0088 (GenBank OK481929), MZB0089 (GenBank OK481928), same data as holotype; 6 exx, 01°03.782N, 120°53.934'E to 01°03.574N, 120°54.032'E, 970–1140 m, 06-VII-2018, beaten; 14 exx, 01°03.574'N, 120°54.032'E to 01°03.157'N, 120°54.195'E, 1100–1120 m, 06-VII-2018, beaten; 7 exx, 01°03.574'N, 120°54.032'E to 01°03.512'N, 120°54.054'E, 1100–1120 m, 06-VII-2018, beaten; 1 ex, MZB0059 (GenBank OK481956), 01°03.512'N, 120°54.054'E, 1100–1200 m, 13-VII-2018, sifted; 7 exx, 01°03.512'N, 120°54.054'E, 1100–1200 m, 06-VII-2018, beaten; 50 exx, MZB0051 (GenBank OK481964), 01°03.574'N, 120°54.032'E to 01°03.181'N, 120°54.607'E, 1100–1400 m, 07-VII-2018, beaten; 15 exx, 01°03.697'N, 120°53.991'E, 1030 m, 06-VII-2018, sifted; 3 exx, MZB0276 (GenBank OK481780), 01°03.412'N, 120°54.126'E to 01°03.241'N, 120°54.328'E, 1200–1300 m, 07-VII-2018, beaten; 3 exx, MZB0049 (GenBank OK481966), MZB0269 (GenBank OK481787), MZB0270 (GenBank OK481786), 01°03.782'N, 120°53.934'E to 01°02.977'N, 120°55.001'E, 1250–1750 m, 11-VII-2018, beaten.

#### Distribution.

C-Sulawesi Prov. (Mt Dako). Elevation 830–1250 m.

#### Biology.

On foliage in montane forests.

#### Etymology.

This epithet is a Latinized adjective based on Toli-Toli regency.

#### Notes.

*Trigonopterustolitoliensis* sp. nov. was coded as “*Trigonopterus* sp. 1186”. The species belongs to the *T.palopensis*-group. It is closely related to *T.puspoi* sp. nov. from which it differs by the setose brushes on the dorsal surface of the penis, the scaling of the metatibia, and a 16.4–18.1% p-distance of its *cox1* sequence.

### 27. Trigonopterus tounensis sp. nov.

Taxon classificationAnimaliaColeopteraCurculionidae

32F84BDA-725F-570D-B822-527165AAA787

http://zoobank.org/B5125BF4-0D58-495C-B570-1B0717AF4809

#### Diagnostic description.

**Figure 27.**
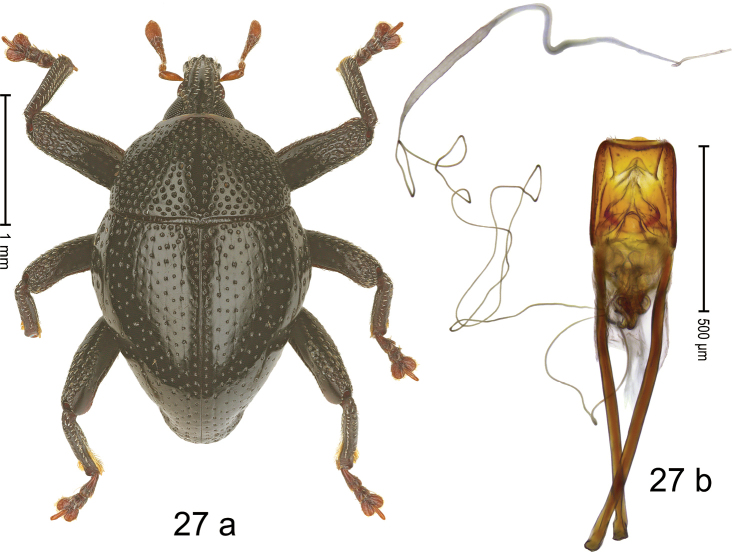
*Trigonopterustounensis* sp. nov., holotype **a** habitus **b** penis.

***Holotype*. Male** (Fig. 27a). Length 2.58 mm. Color of antennae ferruginous; remainder black. Body subovate; in dorsal aspect with weak constriction between pronotum and elytron; in profile dorsally convex. Rostrum dorsally with median costa and pair of submedian ridges; intervening furrows each with sparse row of erect scales; epistome subglabrous with sparse suberect setae, posteriorly with five denticles. Pronotum with disk densely punctate with coarse punctures; almost reticulate, interspaces subglabrous; each puncture containing single, minute seta; medially with impunctate line. Elytra densely irregularly punctate with small punctures; striae indistinct; interspaces between punctures subglabrous; striae 7–9 with larger punctures; stria 8 along humerus with seven large, coarse punctures. Femora edentate, anteroventral ridge simple; anterior surface densely coarsely punctate, each puncture with suberect scale. Metafemur with dorsoposterior edge weakly denticulate; subapically with stridulatory patch; dorsally with rows of silvery scales. Posterior face of metatibia in apical 1/2 with dense yellowish setae. Abdominal ventrites 1–2 concave, densely punctate with coarse punctures and sparse erect scales, sublaterally each with blunt tooth; in profile ventrite 2 subangularly projecting; ventrite 5 densely coarsely punctate, with median impression. Penis (Fig. 27b) with sides of body subparallel; apex with short median angulate extension, with sparse setae; apodemes 2.9× as long as body; transfer apparatus complex; ductus ejaculatorius with distinct bulbus. **Intraspecific variation**. Length 2.38–2.58 mm. Female rostrum flat, dorsally with median costa and row of punctures, and pair of submedian costae; epistome simple, subglabrous with sparse punctures and suberect setae. Female abdominal ventrites 1–2 with shallow impression, surface with sparse coarse punctures; ventrite 5 flat, with dense punctures and suberect setae.

#### Material examined.

***Holotype*** (MZB, Cole.173.079): MZB0110 (GenBank OK481910), Indonesia, C-Sulawesi, Tojo Una-Una, Ulubongka, Mire, Gn. Katopasa, 01°11.31'S, 121°27.348'E, 1380 m, 23–24-VII-2017, beaten. ***Paratype*** (MZB, SMNK): 3 exx, MZB0111 (GenBank OK481909), same data as holotype.

#### Distribution.

C-Sulawesi Prov. (Mt Katopasa). Elevation ca. 1380 m.

#### Biology.

On foliage in montane forest.

#### Etymology.

This epithet is a Latinized adjective based on the Indonesian abbreviation of Tojo Una-Una “Touna” and refers to the type locality.

#### Notes.

*Trigonopterustounensis*, sp. nov. was coded as “*Trigonopterus* sp. 1115” (Narakusumo et al. 2020). This species belongs to the *T.posoensis*-group. It is closely related to *T.obelix* Riedel, which differs by 15.2–16% *cox1* p-distance.

### 28. Trigonopterus unyil sp. nov.

Taxon classificationAnimaliaColeopteraCurculionidae

0D0DB7A4-737B-5A8D-B948-DD4C0754B0E7

http://zoobank.org/74F11B0E-6885-4F20-8886-7484D5C6EF03

#### Diagnostic description.

**Figure 28.**
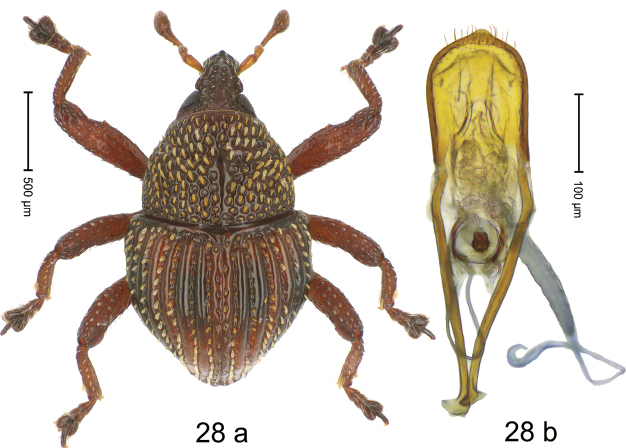
*Trigonopterusunyil* sp. nov., holotype **a** habitus **b** penis.

***Holotype*. Male** (Fig. 28a). Length 1.52 mm. Color largely ferruginous; thorax, sides of elytra and patch at the middle of intervals 2–3 black. Body subovate; in dorsal aspect and in profile with moderate constriction between pronotum and elytron. Rostrum dorsally with dense coarse punctures, areolate-reticulate; with sparse suberect setae; epistome, subglabrous, apically with sparse setae, posteriorly with transverse angulate ridge forming median denticle. Pronotum subapically with weak constriction; disk coarsely punctate, reticulate; each puncture bearing a suberect, clavate, apicad directed, yellowish scale; medially with subglabrous costa, subapically shortened. Elytra with striae marked by rows of suberect subclavate scales; intervals costate, glabrous; basal margin bordered by simple ridge. Femora dentate; anterior surface dull, rugose, but without distinct punctures; with sparse suberect scales. Metafemur dorsally rounded; subapically with stridulatory patch. Abdominal ventrites 1–2 flat, dull, with coarse punctures, with sparse suberect scales; ventrite 5 flat, microreticulate, dull. Penis (Fig. 28b) with sides of body subparallel, towards apex rounded, medially pointed, with sparse setae; apodemes 2.1× as long as body of penis; transfer apparatus denticulate, held by pair of brace-shaped sclerites; ductus ejaculatorius with distinct bulbus. ***Intraspecific variation***. Length 1.38–1.70. Female rostrum subglabrous, with submedian rows of punctures.

**Figure 29.**
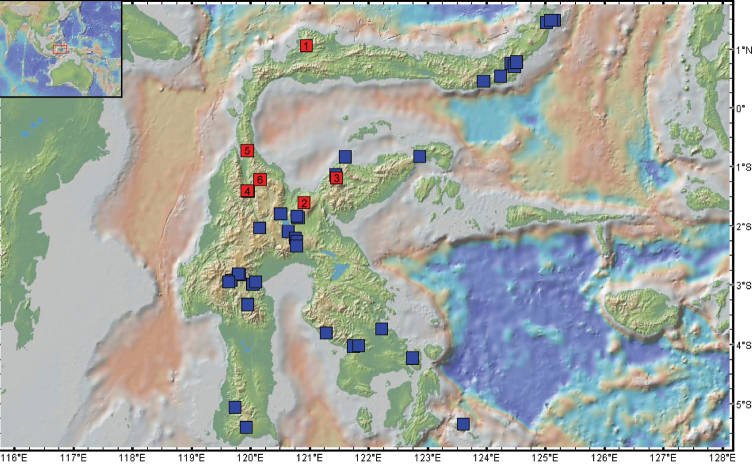
Map of *Trigonopterus* records across Sulawesi and adjacent islands. Blue dots from Riedel and Narakusumo (2019); white dots from present study. Prepared using GeoMapApp (www.geomapapp.org; Ryan et al. 2009); 1 = Mt Dako (*T.acutus* sp. nov., *T.ancora* sp. nov., *T.arcanus* sp. nov., *T.corona* sp. nov., *T.dakoensis* sp. nov., *T.daun* sp. nov., *T.gundala* sp. nov., *T.hoppla* sp. nov., *T.moduai* sp. nov., *T.mons* sp. nov., *T.paramoduai* sp. nov., *T.puspoi* sp. nov., *T.rosichoni* sp. nov., *T.rubidus* sp. nov., *T.sutrisnoi* sp. nov., *T.tanah* sp. nov., *T.tolitoliensis* sp. nov.; 2 = Mt Pompangeo (*T.ewok* sp. nov., *T.kakimerah* sp. nov., *T.matakensis* sp. nov., *T.pomberimbensis* sp. nov., *T.pompangeensis* sp. nov., *T.tejokusumoi* sp. nov., *T.unyil* sp. nov.); 3 = Mt Katopasa (*T.katopasensis* sp. nov., *T.tounensis* sp. nov.); 4 = Mt Torompupu (*T.sarinoi* sp. nov.); 5 = Palu (*T.toboliensis* sp. nov.); 6 = Palolo (*T.toboliensis* sp. nov.).

#### Material examined.

***Holotype*** (MZB, Cole.173.080): MZB0134 (GenBank OK481900), Indonesia, C-Sulawesi, Tojo Una-Una, Matako, Gn. Pompangeo, 01°35.235'S, 120°55.679'E to 01°35.258'S, 120°55.588'E, 1900 m, 27-II-2020, sifted. ***Paratypes*** (MZB, SMNK): Indonesia, C-Sulawesi, Tojo Una-Una, Matako, Gn. Pompangeo: 6 exx, MZB0135 (GenBank OK481899), same data as holotype; 4 exx, MZB0136 (GenBank OK481898), 01°35.197'S, 120°55.658'E to 01°35.127'S, 120°55.622'E, 2000 m, 01-III-2020, sifted.

#### Distribution.

C-Sulawesi Prov. (Mt Pompangeo). Elevation ca. 1900–2000 m.

#### Biology.

In leaf litter of montane forest.

#### Etymology.

This epithet is a noun in apposition based on the Indonesian hand puppet character from “Si Unyil” TV series.

#### Notes.

*Trigonopterusunyil* sp. nov. was coded as “*Trigonopterus* sp. 1196”. Presumably, this species belongs to the *T.nanus*-group.

## Discussion

Relatively short field trips to two mountains in Sulawesi (i.e. five days at Mt Pompangeo and 14 days at Mt Dako) resulted in the discovery of 25 out of 28 *Trigonopterus* species newly described herein. Only a single species, *T.toboliensis* sp. nov. from Palu, respectively Palolo had been collected earlier. This exemplifies what great positive impact dedicated field work can have to species-discovery of neglected arthropod taxa, especially in montane areas. While the more conspicuous species of birds, mammals, butterflies, larger beetles or plants may have been collected on earlier surveys (Bebber et al. 2010; Troudet et al. 2017), the great majority of small-sized beetles of less than 3 mm body size is usually neglected and not sufficiently represented in unidentified museum collections.

It is noteworthy that in some localities pairs of sister species have been discovered: *T.moduai* sp. nov., and *T.paramoduai* sp. nov. are both found in narrowly separated elevation zones of Mt Dako. Although genetically very close (3.20–4.11% *cox1* p-distance) they are very distinct morphologically. Other such species pairs are morphologically very hard or impossible to separate, but very divergent genetically, i.e. *T.matakensis* sp. nov. and *T.pompangeensis* sp. nov. from Mt Pompangeo or the allopatric *T.ovatulus* Riedel and *T.pseudovatulus* Riedel from mountains north of Lake Poso (Riedel and Narakusumo 2019). It would be interesting to explore factors of their speciation in detail.

Mt Dako is a nature reserve, and forests above 800 m are largely intact. On the other hand, Mt Pompangeo is without any conservation status and has been logged extensively between 1970 m and 2000 m. Patches of rainforest remaining in the steeper areas are still at risk being affected by regular forest fires. Both Mt Dako and Mt Pompangeo harbour endemic *Trigonopterus* species, and presumably additional ones could be discovered if longer field trips are conducted in the remaining forest patches. These forest patches among the Sulawesi rainforest still hold the largely unknown diversity of *Trigonopterus* and other arthropod species. They should be of greater concern to conservation despite or rather because of their fragmentation.

## Provisional catalogue of species groups of *Trigonopterus* in Sulawesi

**subgenus Mimidotasia Voss**: *T.pauper* Riedel, *T.luwukensis* Riedel, *T.selayarensis* Riedel, *T.wangiwangiensis* Riedel.

***T.abnormis*-group**: *T.abnormis* Riedel, *T.kolakensis* Riedel

***T.analis*-group**: *T.analis* Riedel, *T.pagaranganensis* Riedel, *T.pseudanalis* Riedel

***T.arachnobas*-group**: *T.arachnobas* Riedel, *T.darwini* Riedel, *T.gracilipes* Riedel, *T.idefix* Riedel, *T.moduai* sp. nov., *T.paramoduai* sp. nov., *T.pumilus* Riedel, *T.rudis* Riedel, *T.tanah* sp. nov., *T.tenuipes* Riedel.

***T.barbipes*-group**: *T.barbipes* Riedel, *T.ejaculatorius* Riedel, *T.katopasensis* sp. nov., *T.pomberimbensis* sp. nov., *T.tejokusumoi* sp. nov., *T.vicinus* Riedel, *T.viduus* Riedel.

***T.bornensis*-group**: *T.rotundatus* Riedel.

***T.collaris*-group**: *T.collaris* Riedel, *T.klabatensis* Riedel, *T.paracollaris* Riedel.

***T.crenulatus*-group**: *T.costatulus* Riedel, *T.crenulatus* Riedel, *T.humilis* Riedel, *T.squalidulus* Riedel.

***T.curtus*-group**: *T.procurtus* Riedel, *T.pseudosimulans* Riedel, *T.scitulus* Riedel.

***T.dimorphus*-group**: *T.reticulatus* Riedel, *T.scabripes* Riedel, *T.pendolensis* Riedel.

***T.fulvicornis*-group**: *T.celebensis* Riedel, *T.corona* sp. nov., *T.fulvicornis* (Pascoe, 1885), *T.kakimerah* sp. nov., *T.pseudofulvicornis* Riedel, *T.seticnemis* Riedel.

***T.honestus*-group**: *T.inhonestus* Riedel.

***T.incendium*-group**: *T.incendium* Riedel.

***T.impressicollis*-group**: *T.adspersus* Riedel, *T.castaneipennis* Riedel, *T.ewok* sp. nov., *T.impressicollis* Riedel, *T.mesai* Riedel, *T.serripes* Riedel, *T.suturatus* Riedel.

***T.laevigatus*-group**: *T.invalidus* Riedel, *T.jasminae* Riedel, *T.laevigatus* Riedel.

***T.lampros*-group**: *T.artemis* Riedel, *T.carinirostris* Riedel, *T.curvipes* Riedel, *T.lampros* Riedel, *T.sarinoi* sp. nov., *T.yoda* Riedel.

***T.manadensis*-group**: *T.ambangensis* Riedel, *T.armipes* Riedel, *T.cirripes* Riedel, *T.dakoensis* sp. nov., *T.heberti* Riedel, *T.mahawuensis* Riedel, *T.manadensis* Riedel, *T.modoindingensis* Riedel, *T.pseudomanadensis* Riedel, *T.volcanorum* Riedel.

***T.minahassae*-group**: *T.indigenus* Riedel, *T.minahassae* Riedel, *T.prismae* Riedel, *T.sampunensis* Riedel, *T.silvicola* Riedel.

***T.nanus*-group**: *T.hirsutus* Riedel, *T.nanus* Riedel, *T.unyil* sp. nov..

***T.nitidulus*-group**: *T.cricki* Riedel, *T.nitidulus* Riedel.

***T.ovalipunctatus*-group**: *T.lompobattangensis* Riedel, *T.matakensis* sp. nov., *T.ovalipunctatus* Riedel, *T.pompangeensis* sp. nov., *T.pseudovalipunctatus* Riedel.

***T.ovatulus*-group**: *T.arcanus* sp. nov., *T.ovatulus* Riedel, *T.pseudovatulus* Riedel.

***T.palopensis*-group**: *T.asterix* Riedel, *T.fuscipes* Riedel, *T.kotamobagensis* Riedel, *T.latipennis* Riedel, *T.matalibaruensis* Riedel, *T.moatensis* Riedel, *T.palopensis* Riedel, *T.puspoi* sp. nov., *T.rhombiformis* Riedel, *T.rufipes* Riedel, *T.tolitoliensis* sp. nov..

***T.politus*-group**: *T.allotopus* Riedel, *T.pseudallotopus* Riedel.

***T.posoensis*-group**: *T.obelix* Riedel, *T.posoensis* Riedel, *T.tounensis* sp. nov..

***T.relictus*-group**: *T.mangkutanensis* Riedel

***T.rotundulus*-group**: *T.rotundulus* Riedel, *T.watsoni* Riedel

***T.saltator*-group**: *T.bonthainensis* Riedel

***T.satyrus*-group**: *T.ancora* sp. nov., *T.gundala* sp. nov., *T.mons* sp. nov., *T.rosichoni* sp. nov., *T.satyrus* Riedel.

***T.sampuragensis*-group**: *T.sampuragensis* Riedel.

***T.tatorensis*-group**: *T.acutus* sp. nov., *T.daun* sp. nov., *T.hoppla* sp. nov., *T.hypocrita* Riedel, *T.incognitus* Riedel, *T.rubidus* sp. nov., *T.sulawesiensis* Riedel, *T.tatorensis* Riedel, *T.tomohonensis* Riedel.

***T.toraja*-group**: *T.ampanensis* Riedel, *T.rantepao* Riedel, *T.scaphiformis* Riedel, *T.sutrisnoi* sp. nov., *T.toboliensis* sp. nov., *T.toraja* Riedel.

## Supplementary Material

XML Treatment for Trigonopterus

XML Treatment for Trigonopterus acutus

XML Treatment for Trigonopterus ancora

XML Treatment for Trigonopterus arcanus

XML Treatment for Trigonopterus corona

XML Treatment for Trigonopterus dakoensis

XML Treatment for Trigonopterus daun

XML Treatment for Trigonopterus ewok

XML Treatment for Trigonopterus gundala

XML Treatment for Trigonopterus hoppla

XML Treatment for Trigonopterus kakimerah

XML Treatment for Trigonopterus katopasensis

XML Treatment for Trigonopterus matakensis

XML Treatment for Trigonopterus moduai

XML Treatment for Trigonopterus mons

XML Treatment for Trigonopterus paramoduai

XML Treatment for Trigonopterus pomberimbensis

XML Treatment for Trigonopterus pompangeensis

XML Treatment for Trigonopterus puspoi

XML Treatment for Trigonopterus rosichoni

XML Treatment for Trigonopterus rubidus

XML Treatment for Trigonopterus sarinoi

XML Treatment for Trigonopterus sutrisnoi

XML Treatment for Trigonopterus tanah

XML Treatment for Trigonopterus tejokusumoi

XML Treatment for Trigonopterus toboliensis

XML Treatment for Trigonopterus tolitoliensis

XML Treatment for Trigonopterus tounensis

XML Treatment for Trigonopterus unyil
